# Synthesis of Aromatic Poly(Thioether)s with Phosphine Sulfide Groups for High-Refractive-Index Materials

**DOI:** 10.1021/acsomega.5c13502

**Published:** 2026-04-01

**Authors:** Ryoyu Hifumi, Akiyoshi Takai, Ikuyoshi Tomita

**Affiliations:** Department of Chemical Science and Engineering, School of Materials and Chemical Technology, 13290 Institute of Science Tokyo, Nagatsuta-cho 4259-G1-9, Midori-ku, Yokohama 226-8501, Japan

## Abstract

High-refractive-index polymers are essential for developing high-performance optical components in modern devices, and one effective approach to designing such materials is to incorporate functional groups containing heavy atoms and/or multiple bonds into polymer structures in higher contents. In the present study, we developed a series of aromatic poly­(thioether)­s incorporating phosphine sulfide (P=S) groups, which contain relatively heavy heteroatoms and multiple bonds between them, for high-refractive-index materials. The polymers were synthesized via nucleophilic aromatic substitution (S_N_Ar) reactions involving monomers with the P=S groups or an S_N_Ar reaction involving a monomer with a phosphine oxide (P=O) group and subsequent conversion of the P=O to P=S groups using Lawesson’s reagent. The poly­(thioether)­s containing the P=S groups achieved higher refractive indices, ranging from 1.725 to 1.757 at 589 nm, than the corresponding polymers containing either the P=S or thioether moieties. The refractive indices of these poly­((thio)­ether)­s containing the P=O or P=S groups positively correlate with the heavy heteroatom (S and P) content of the polymers, indicating that the combination of the P=S groups and the thioether linkages effectively enhances the refractive indices of the polymers. The above results highlight the potential of the P=S group for expanding the structural diversity of polymeric optical materials.

## Graphical abstract

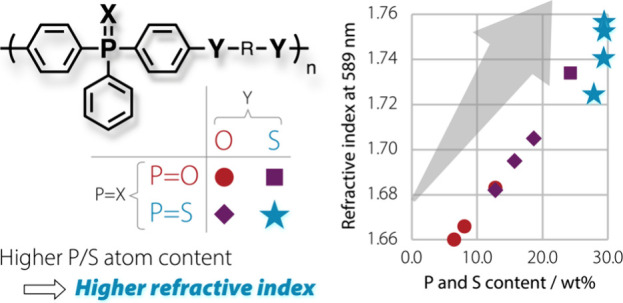

## Introduction

Polymeric materials with high refractive indices have been studied over the past few decades for both fundamental and industrial interests because of their many optical applications, including plastic lenses, optical fibers and waveguides, encapsulants, and optical films and coatings.^1–5^ The higher refractive indices of the polymeric materials can be achieved (i) by increasing the density (decreasing the free volume) of the materials and/or (ii) by introducing functional groups with high molar refractivities (*R*_M_ values) according to the Lorentz–Lorenz eq (eq 1).^1,4,5^

$$\frac{n^{2}-1}{n^{2}+2}=\frac{\rho }{M_{0}}\sum R_{M}$$ 1

where *n*, *ρ*, and *M*_0_ are the refractive index, the density of the material, and the molecular weight of the repeating unit, respectively. Owing to the broader scope of the molecular design compared to approach (i), many types of high-refractive-index polymers have been developed based on approach (ii). The *R*_M_ values of various functional groups have been reported, in which the functional groups with multiple bonds and/or heavier atoms have high values.

The *R*_M_ values of unsaturated structures, such as the alkene (C=C, 6.6 cm^3^ mol^–1^), alkyne (C≡C, 7.2 cm^3^ mol^–1^), and imine (C=N, 6.5 cm^3^ mol^–1^) groups, are higher than that of the saturated aliphatic group (C–C, 4.8 cm^3^ mol^–1^).^1,6^ Aromatic polymers incorporating fused aromatic moieties^7,8^ and/or cardo/spiro-type fluorene^9–12^ structures are widely applied to high-refractive-index materials owing to their high content of the C=C unit. In addition, polymers containing heteroaromatic rings with nitrogen atoms have been reported to exhibit high refractive indices.^13–15^

Among the heteroatom-containing polymers, those with sulfur atoms (*R*_M_ = 7.8 cm^3^ mol^–1^) are extensively studied,^1,5^ such as sulfur-containing vinyl polymers^16,17^ and poly­(imide)­s,^18,19^ poly­(thioether)­s,^14,20–24^ poly­(thiourea)­s,^25^ poly­(dithioacetal)­s,^26^ poly­(trithiocarbonate)­s,^27^ and other sulfur-rich polymers.^28–30^ In addition, high refractive polymers containing heavier atoms, such as chlorine,^31^ bromine,^32–34^ iodine,^32,35^ bismuth,^36^ selenium,^37–42^ and/or tellurium,^39,43^ have been developed.

Phosphorus-containing functional groups also have moderate to high *R*_M_ values, such as phosphine oxide (P=O, 6.5–8.0 cm^3^ mol^–1^)^44,45^ and phosphazene (P=N, 14.4 cm^3^ mol^–1^) groups,^46^ and have been used to develop high refractive polymers.^47^ Shobha et al. and Macdonald et al. independently synthesized poly­(phosphonate)­s with various bisphenol moieties, whose refractive indices were up to 1.66 at 633 nm.^48,49^ Saegusa et al. reported that the refractive index of a bisphenol A-based poly­(ether) with triphenylphosphine oxide moieties was 1.643 at 590 nm,^50^ which was higher than that of a corresponding poly­(ether) with diphenyl sulfone moieties (1.636 at 589 nm).^51^ You and co-workers synthesized a series of aromatic poly­(imide)­s containing thioether and triphenylphosphine oxide moieties with refractive indices ranging from 1.640 to 1.725.^52^ Sun, Fang, and co-workers developed cross-linked materials through thiol–ene reactions involving dithiol compounds and either tri­(4-vinylphenyl) phosphate or hexa­(4-vinylphenoxy)­cyclotriphosphazene, whose refractive indices were reported to be 1.698–1.721 and 1.673–1.706, respectively.^53,54^ Olshavsky and Allcock reported linear poly­(phosphazene)­s possessing various aryloxy pendant groups with high refractive indices of up to 1.755.^46,55^

A phosphine sulfide (P=S) group has a high *R*_M_ value of 14.8–15.4 cm^3^ mol^–1^ due to the presence of the period 3 elements and a multiple bond between the P and S atoms, as reported in the studies of liquid organic phosphorus compounds.^44,45^ However, compared to the other phosphorus-containing polymers, limited examples of high-refractive materials containing the P=S groups were reported. Manners and co-workers synthesized poly­(ferrocene) derivatives with the P=S moieties as linkers via the anionic ring-opening polymerization of [1]­phosphaferrocenophane monomers, followed by sulfurization of the phosphine moieties.^56,57^ The resulting materials were reported to have refractive indices of up to 1.723 at 589 nm. Su et al. developed cross-linked materials with refractive indices ranging from 1.676 to 1.732 through thiol–ene reactions involving trivinylphosphine sulfide and dithiol compounds.^41^ We have reported two types of linear polymers containing the P=S groups, poly­(thiophosphonate)­s and aromatic poly­(ether)­s containing the P=S groups, whose refractive indices are up to 1.687 and 1.691, respectively, at 589 nm and are higher than those of the corresponding polymers containing the P=O groups.^51,58^

In order to provide further high-refractive-index materials utilizing the P=S groups, it would be promising to incorporate thioether moieties as linkers instead of the aliphatic or ether moieties, which have lower *R*_M_ values and were used in the previous studies.^41,51,58^ Although the synthesis and thermal properties of a poly­(thioether) containing the P=S groups were reported by Mohanty and co-workers, its optical properties have not been described.^59^ Herein, we synthesize a series of P=S group-containing poly­(thioether)­s with various structures to figure out their higher refractive indices compared to those of the corresponding P=O group-containing poly­((thio)­ether)­s and P=S group-containing poly­(ether)­s (Figure 1).

**Figure 1:**
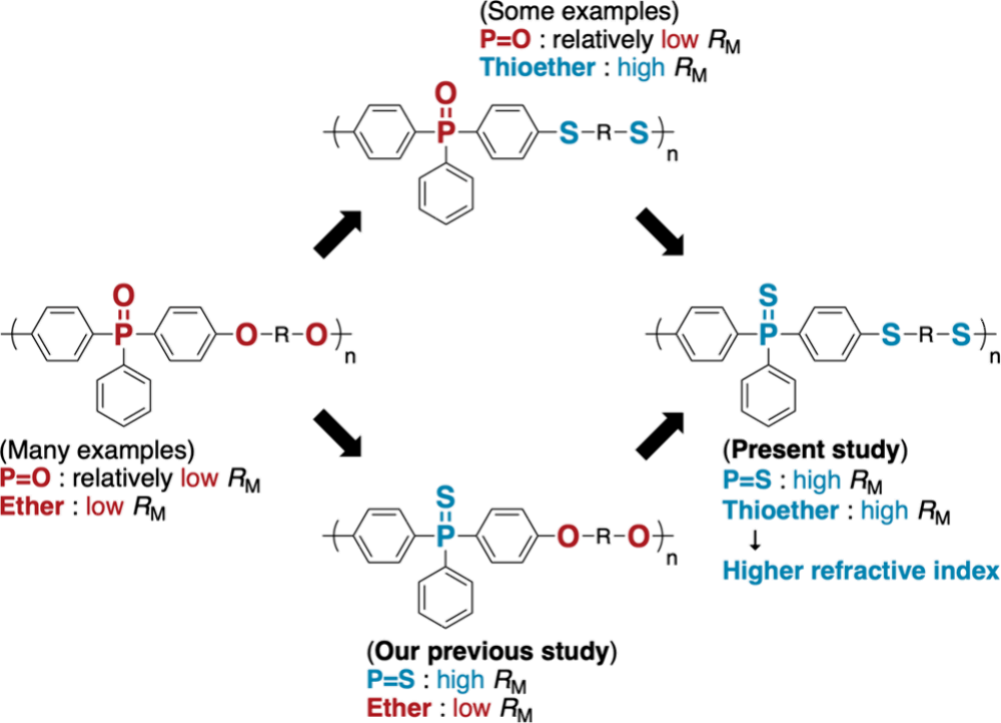
Molecular design of aromatic poly­(thioether)­s containing P=S groups for high-refractive-index materials.

## Methods

### Monomer Synthesis

Bis­(4-fluorophenyl)­phenylphosphine oxide and bis­(4-fluorophenyl)­phenylphosphine sulfide were synthesized according to the methods described in our previous publications.^51,60^

Bis­(4-mercaptophenyl)­phenylphosphine sulfide was synthesized according to the method employed for the synthesis of bis­(4-mercaptophenyl) sulfone.^12^ Bis­(4-fluorophenyl)­phenylphosphine sulfide (1.00 g, 3.03 mmol) was added to a solution of NaSH·*n*H_2_O (1.01 g, 65 wt %, 11.7 mmol, 3.86 equiv) in *N*,*N*-dimethylformamide (10 mL) under nitrogen, and the resulting mixture was stirred at 150 °C for 5 h. After the reaction mixture was cooled to ambient temperature, insoluble salts were removed by filtration. The filtrate was poured into dilute aqueous HCl to form a precipitate, and the resulting solid was recovered by filtration. The solid was dissolved in aqueous NaOH, and the insoluble part was removed by filtration. The filtrate was poured into aqueous HCl to give bis­(4-mercaptophenyl)­phenylphosphine sulfide as a white solid (0.858 g, 2.39 mmol, 78.9% yield). Melting point: 88.6–89.6 °C. ^1^H NMR (400 MHz, CDCl_3_) δ 7.68 (m, 2H), 7.57–7.50 (5H), 7.45 (m, 2H), 7.30 (m, 4H), 3.59 (s, 2H) ppm. ^13^C­{^1^H} NMR (101 MHz, CDCl_3_) δ 137.1 (d, ^4^ *J*_C–P_ = 3.4 Hz), 132.8 (d, *J*_C–P_ = 11.6 Hz), 132.7 (d, ^1^ *J*_C–P_ = 86.7 Hz), 132.1 (d, *J*_C–P_ = 10.6 Hz), 131.7 (d, ^4^ *J*_C–P_ = 2.4 Hz), 129.3 (d, ^1^ *J*_C–P_ = 88.1 Hz), 128.6 (d, *J*_C–P_ = 12.5 Hz), 128.3 (d, *J*_C–P_ = 13.0 Hz) ppm. ^31^P­{^1^H} NMR (162 MHz, CDCl_3_) δ 43.0 (s) ppm. HRMS (ESI) *m*/*z*: [M – H]^−^ calcd for C_18_H_14_PS_3_, 357.0001; found, 356.9993.

### Polymer Synthesis

Polymerization through nucleophilic aromatic substitution reactions of arylene difluoride monomers with bis­(thio)­phenol monomers, as well as the conversion of the P=O to P=S groups in the polymers, were performed according to the procedures described in our previous publications.^51,60,61^ Typical examples are as follows:

Bis­(4-fluorophenyl)­phenylphosphine oxide (3.77 g, 12.0 mmol, 1 equiv) and bis­(4-mercaptophenyl) sulfide (2.94 g, 11.7 mmol, 0.975 equiv) were reacted in the presence of K_2_CO_3_ (2.74 g, 19.8 mmol, 1.65 equiv) in 1-methyl-2-pyrrolidone (NMP, 20 mL) at 130 °C for 1 h and then at 180 °C for 10.5 h under nitrogen. After cooling to ambient temperature, the reaction mixture was diluted with tetrahydrofuran (THF) and filtered through Kiriyama No. 5B filter paper to remove insoluble salts. The filtrate was poured into dilute aqueous HCl, and the resulting solid was washed with deionized water (twice) and acetone (3 times). After dissolving the obtained solid in THF, the solution was poured into deionized water, and the resulting solid was washed with deionized water (3 times) and dried in a vacuum oven at 120 °C for 5 h. The polymer (**P5**) was obtained as a fibrous solid (6.00 g, 96.1% yield). Number-average molecular weight (*M*_n_) = 22000 and weight-average molecular weight (*M*_w_) = 54700 (size exclusion chromatography (SEC) in NMP with LiBr). ^1^H NMR (400 MHz, CDCl_3_) δ 7.65–7.60 (2H), 7.55–7.48 (5H), 7.46–7.42 (2H), 7.38–7.29 (8H), 7.26–7.23 (4H) ppm. ^13^C­{^1^H} NMR (101 MHz, CDCl_3_) δ 142.5 (d, ^4^ *J*_C–P_ = 2.9 Hz), 135.9, 133.9, 132.6 (d, *J*_C–P_ = 10.6 Hz), 132.1 (d, ^4^ *J*_C–P_ = 2.4 Hz), 131.9 (d, *J*_C–P_ = 10.1 Hz), 131.8, 131.7, 131.5, 129.9 (d, ^1^ *J*_C–P_ = 106.4 Hz), 128.6 (d, *J*_C–P_ = 12.0 Hz), 128.2 (d, *J*_C–P_ = 12.5 Hz) ppm. ^31^P­{^1^H} NMR (162 MHz, CDCl_3_) δ 29.0 (s) ppm.

The poly­(thioether) with phosphine oxide groups (**P5**, 3.04 g, 5.79 mmol, 1 equiv) and Lawesson’s reagent (2.00 g, 4.94 mmol, 0.853 equiv) were reacted in toluene (88 mL) under reflux for 24.5 h under nitrogen. The reaction mixture was cooled to ambient temperature, and the volatile fraction was evaporated under reduced pressure. After the residue was dissolved in CHCl_3_, the solution was poured into acetone, and the resulting solid was washed with acetone (twice). The solid was dissolved in THF, and the resulting solution was poured into methanol. The resulting solid was washed with methanol (3 times) and dried in a vacuum oven at 120 °C for 10 h. The polymer (**P6**) was obtained as a fibrous solid (2.87 g, 91.7% yield). *M*_n_ = 28600 and *M*_w_ = 61900 (SEC in NMP with LiBr). ^1^H NMR (400 MHz, CDCl_3_) δ 7.70–7.65 (2H), 7.58–7.47 (5H), 7.44–7.28 (10H), 7.21 (dd, ^3^ *J*_H–H_ = 8.4 Hz, *J* = 2.3 Hz, 4H) ppm. ^13^C­{^1^H} NMR (101 MHz, CDCl_3_) δ 142.3 (d, ^4^ *J*_C–P_ = 3.4 Hz), 136.0, 134.0, 132.7 (d, *J*_C–P_ = 11.1 Hz), 132.4 (d, ^1^ *J*_C–P_ = 85.7 Hz), 132.1 (d, *J*_C–P_ = 10.6 Hz), 131.8, 131.7 (d, ^4^ *J*_C–P_ = 2.9 Hz), 131.6, 130.3 (d, ^1^ *J*_C–P_ = 87.2 Hz), 128.6 (d, *J*_C–P_ = 12.5 Hz), 128.1 (d, *J*_C–P_ = 13.0 Hz) ppm. ^31^P­{^1^H} NMR (162 MHz, CDCl_3_) δ 42.9 (s) ppm.

The other polymers were likewise synthesized, and their characterization data are shown in the Supporting Information.

## Results and Discussion

### Polymer Synthesis and Characterization

Polymerizations were performed based on nucleophilic aromatic substitution reactions of arylene difluorides having P=O, P=S, or sulfone (SO_2_) moieties as activating groups with bis­(thio)­phenols to give aromatic poly­((thio)­ether)­s (Scheme 1). The synthesis of the P=O group-containing polymers (**P1**, **P3**, and **P5**) has already been described, in which their number- and weight-average molecular weights (*M*_n_ and *M*_w_ values, respectively) are in the range of 19100–25900 and 54700–106300, respectively (Table 1).^61^ By a similar method, **P7** was successfully obtained, whose *M*_n_ and *M*_w_ values are 12300 and 28400, respectively. The reaction of bis­(4-fluorophenyl)­phenylphosphine sulfide with 1,3-dimercaptobenzene could also give the polymer (**P9**) with *M*_n_ = 17400 and *M*_w_ = 77200. The reaction of bis­(4-mercaptophenyl)­phenylphosphine sulfide with bis­(4-fluorophenyl) sulfone or bis­(4-fluorophenyl)­phenylphosphine sulfide gave the polymers (**P10** or **P11**, respectively) with moderate molecular weights of *M*_n_ = 7900–10600 and *M*_w_ = 17200–18000, presumably because the electron-withdrawing P=S group reduces the nucleophilic reactivity of the thiol groups. The polymer structures have been fully characterized by ^1^H, ^13^C, and ^31^P nuclear magnetic resonance (NMR) spectroscopy, as shown in the Supporting Information. In the ^31^P NMR spectra, **P7** exhibited only a signal corresponding to the P=O group at 29.0 ppm (Figure S31), while **P9**–**P11** exhibited only signals attributable to the P=S group in the range of 42.7–43.0 ppm (Figures S39, S43, and S47). These results indicate that both the P=O and P=S groups were preserved under polymerization conditions.

**Scheme 1:**
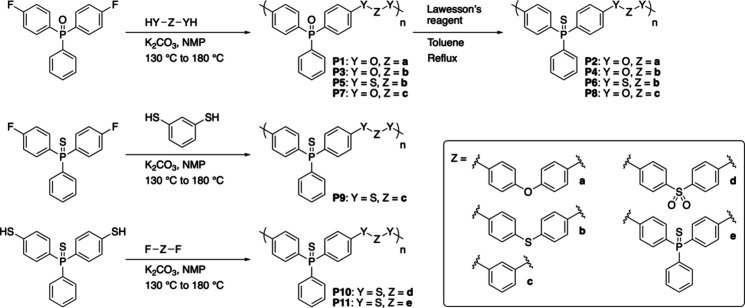
Synthetic Schemes for Aromatic Poly­((Thio)­Ether)­s with P=O or P=S Groups (**P1**–**P11**)­^a^

a NMP = 1-methyl-2-pyrrolidone, Lawesson’s reagent = 2,4-bis­(4-methoxyphenyl)-1,3,2,4-dithiadiphosphetane-2,4-dithione.

**Table 1:** Molecular Weights and Thermal and Optical Properties of Aromatic Poly­((Thio)­Ether)­s with P=O or P=S Groups (**P1**–**P11**)

	Molecular weights		Thermal properties		Optical properties			
Polymers	*M*_n_ ^a^	*M*_w_ ^a^	*T*_d5_ (°C)^b^	*T*_g_ (°C)^c^	*n*_F_ ^d^	*n*_D_ ^d^	*n*_C_ ^d^	*ν*_D_ ^e^
**P1**	25900	77800	558	189^f^	1.682	1.660	1.652	22.0
**P2**	43300	88500	520	194^f^	1.706	1.682	1.673	20.7
**P3**	19100	106300	539	180^f^	1.706	1.683	1.674	21.3
**P4**	27800	174300	503	177^f^	1.730	1.705	1.696	20.7
**P5**	22000	54700	499^g^	175^f^ ^,^ ^g^	1.766	1.734	1.722	16.7
**P6**	28600	61900	476	181^f^ ^,^ ^h^	1.793	1.757	1.747	16.5
**P7**	12300	28400	550	179	1.686	1.666	1.658	23.8
**P8**	28700	51300	519	170	1.717	1.695	1.684	21.1
**P9**	17400	77200	479	175	1.785	1.753	1.740	16.7
**P10**	10600	18000	448	228	1.749	1.725	1.712	19.6
**P11**	7900	17200	464	247	1.756	1.741	1.721	21.2

a Measured by size exclusion chromatography (SEC) calibrated with polystyrene standards in 1-methyl-2-pyrrolidone with LiBr (10 mM) or in CHCl_3_. *M*_n_ and *M*_w_ are the number- and weight-average molecular weights, respectively.

b Temperature at 5% weight loss measured under nitrogen.

c Glass transition temperature measured under nitrogen.

d *n*_F_, *n*_D_, and *n*_C_ values are refractive indices at 486, 589, and 656 nm, respectively.

e *ν*_D_ value is the Abbe number defined as (*n*_D_ – 1)/(*n*_F_ – *n*_C_).

f The *T*_g_ values of **P1**–**P6** were reported in our previous paper.^61^

g The *T*_d5_ and *T*_g_ values of **P5** were reported to be 500 and 181 °C, respectively.^62^

h The *T*_g_ value of **P6** was reported to be 180 °C.^59^

The polymers containing the P=S groups could also be synthesized from the corresponding polymers containing the P=O groups via reactions with Lawesson’s reagent (Scheme 1), as described in our previous papers, including the synthesis of **P2**, **P4**, and **P6**.^61^ According to this method, **P7** was converted into the P=S group-containing polymer (**P8**) with *M*_n_ = 28700 and *M*_w_ = 51300 (Table 1). In the ^31^P NMR spectrum of **P8**, there was no peak corresponding to the phosphorus atoms of the P=O groups (∼ 29 ppm), and only the peak corresponding to the P=S groups was observed at 42.4 ppm, indicating the quantitative conversion of the P=O to P=S groups (Figure S35).

The polymers (**P1**–**P9**), which possess sufficiently high molecular weights, afforded tough and free-standing films when prepared by casting from their CH_2_Cl_2_ solutions.^51^ In contrast, **P10** and **P11** yielded brittle films, which can likely be attributed to their relatively lower molecular weights.

### Thermal Properties

All of the poly­((thio)­ether)­s synthesized herein exhibited excellent thermal stability and high glass transition temperatures (*T*_g_ values) in thermogravimetric analysis (TGA) and differential scanning calorimetry (DSC) measurements (Table 1 and Figure 2). Compared with the corresponding polymers containing the P=O groups (**P1**, **P3**, **P5**, and **P7**), the polymers containing the P=S groups (**P2**, **P4**, **P6**, and **P8**) exhibited slightly lower 5% weight loss temperatures (*T*_d5_ values) by approximately 30 °C. Importantly, the polymers (**P1**–**P11**) have *T*_d5_ values above 440 °C (Figure 2a), which are sufficient for the manufacturing and practical use of optical components. In the DSC measurements, a series of the poly­((thio)­ether)­s with similar structures (**P1**–**P9**) showed similar *T*_g_ values in the range of 170–194 °C (Figure 2b). The polymer with SO_2_ moieties (**P10**) exhibited a *T*_g_ value of 228 °C, which is higher than that of the corresponding polymer with sulfide moieties (**P6**). The polymer (**P11**) exhibited the highest *T*_g_ value in this study (247 °C), presumably owing to the high content of bulky triphenylphosphine sulfide moieties.

**Figure 2:**
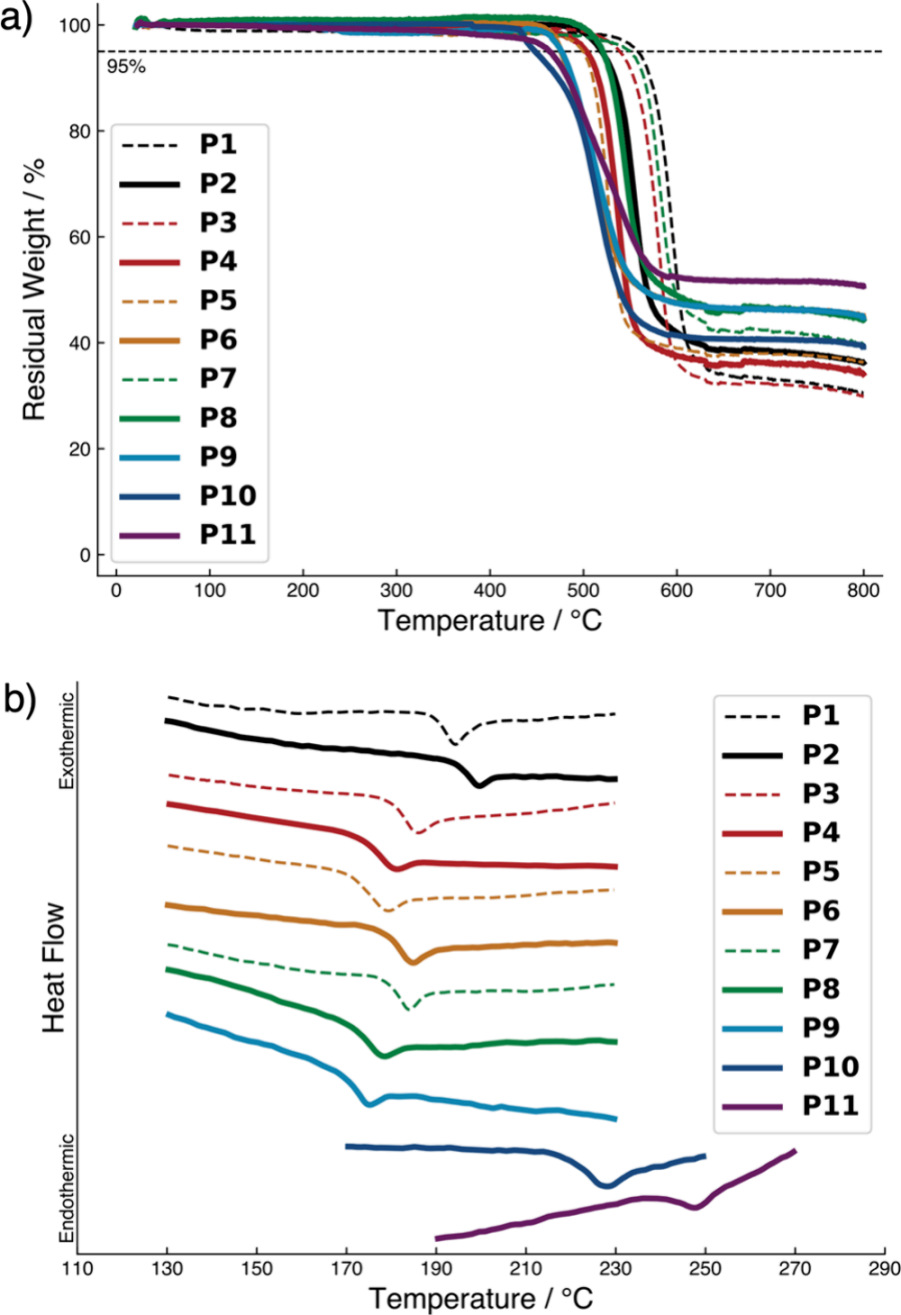
(a) TGA curves of **P1**–**P11** under nitrogen. (b) DSC curves of **P1**–**P11** under nitrogen. The DSC curves of **P1**–**P6** were reported in our previous paper. (Adapted with permission from Hifumi, R.; Tomita, I. Synthesis and Dielectric Properties of Aromatic Poly­(thioether)­s with Triphenylphosphine Sulfide Moieties. *J. Netw. Polym. Jpn*. **2024**, *45*, 143–150.^61^ Copyright 2024 Japan Thermosetting Plastics Industry Association.)

### Optical Properties

The optical transparency of the poly­(thioether)­s containing the P=S groups (**P6** and **P9**–**P11**) was evaluated using ultraviolet (UV)–vis absorption and transmittance spectra (Figure 3). The CHCl_3_ solutions of the polymers exhibited no absorbance at wavelengths above 360 nm (Figure 3a). Their films have cutoff wavelengths below 356 nm (Figure 3b), which are comparable to the absorption edges observed in the solutions. In addition, the films exhibited transmittance of over 77% in the range of 400–700 nm (Figure 3b) and showed no significant coloration (Figure 3c). These results indicate that the poly­(thioether)­s containing the P=S groups have sufficient transparency for optical applications.

**Figure 3:**
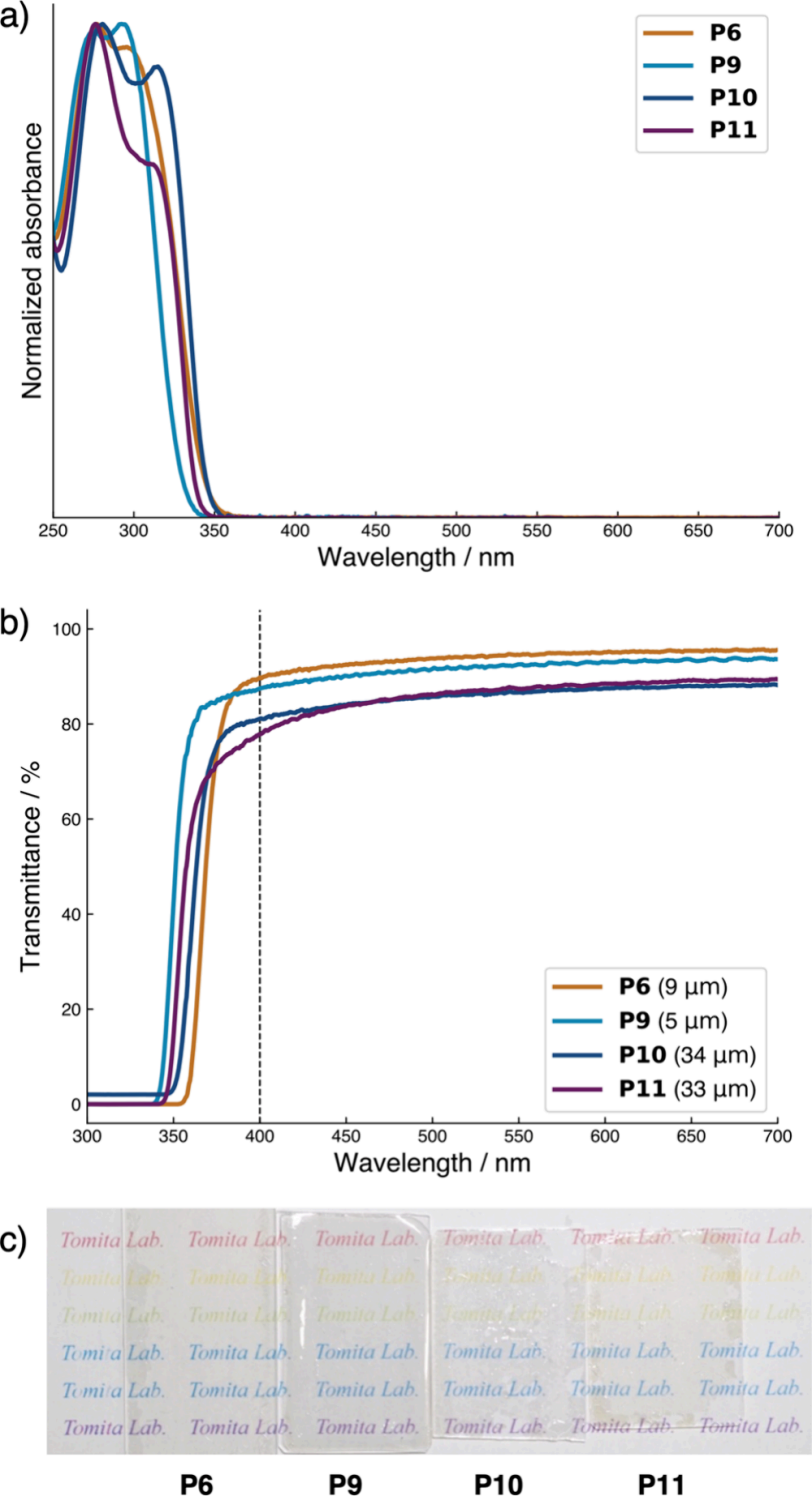
(a) UV–vis absorption spectra in CHCl_3_ solutions, (b) transmittance spectra in film states (film thicknesses are represented in parentheses), and (c) appearance of films on glass substrates for **P6** and **P9**–**P11**.

The refractive indices of the polymers were evaluated at 486 (*n*_F_), 589 (*n*_D_), and 656 (*n*_C_) nm using an Abbe refractometer, and some polymers showed high *n*_D_ values above 1.70 (Table 1 and Figure 4). The *n*_D_ values of the polymers containing the P=S groups (**P2**, **P4**, **P6**, and **P8**) are in the range of 1.682–1.757, which are higher than the respective values of the corresponding polymers containing the P=O groups (**P1**, **P3**, **P5**, and **P7**, *n*_D_ = 1.660–1.734). In addition, the *n*_D_ values of the polymers increased, as the ether moieties in the polymer backbones were replaced by the thioether moieties: **P1** (*n*_D_ = 1.660) < **P3** (1.683) < **P5** (1.734), **P2** (1.682) < **P4** (1.705) < **P6** (1.757), and **P8** (1.695) < **P9** (1.753). The polymer with SO_2_ moieties (**P10**) has a high *n*_D_ value of 1.725, which is, however, slightly lower than that of the thioether type (**P6**), probably due to the large free volume caused by the bulky SO_2_ groups. The polymer with triphenylphosphine sulfide moieties directly linked by the thioether bonds (**P11**) has a high *n*_D_ value of 1.741.

**Figure 4:**
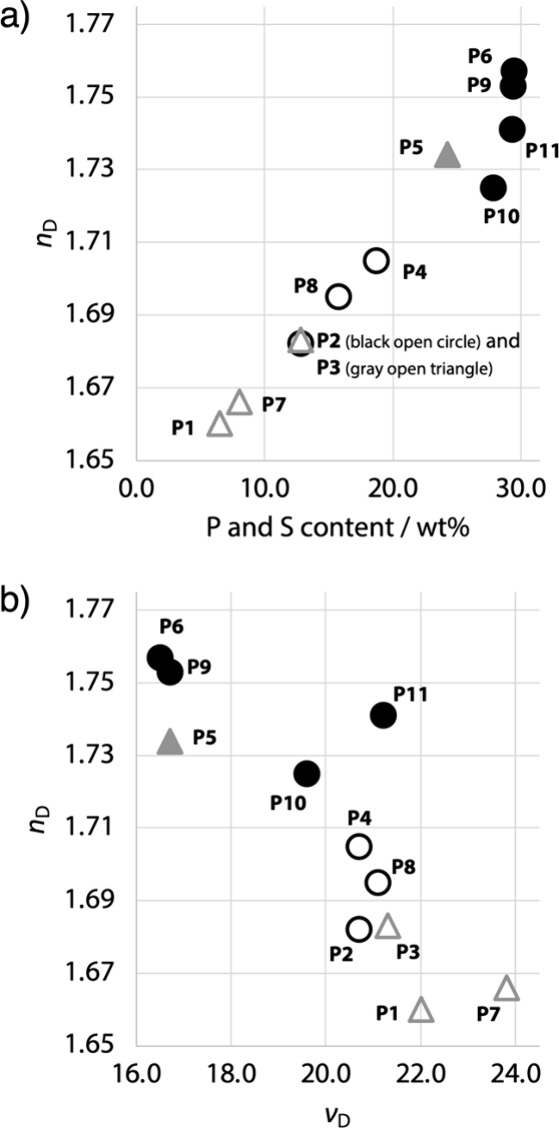
(a) Relationship between the heteroatom (S and P) content and the *n*_D_ values of **P1**–**P11**. (b) *n*_D_ values vs *ν*_D_ values of **P1**–**P11**. Gray open triangles represent the poly­(ether)­s containing the P=O groups (**P1**, **P3**, and **P7**), a gray solid triangle represents the poly­(thioether) containing the P=O groups (**P5**), black open circles represent the poly­(ether)­s containing the P=S groups (**P2**, **P4**, and **P8**), and black solid circles represent the poly­(thioether)­s containing the P=S groups (**P6** and **P9**–**P11**).

The densities of the polymers (**P1**–**P6**) were measured to clarify the influence of the density on refractive indices (Table S1). The polymers containing the P=O groups (**P1**, **P3**, and **P5**) exhibited densities in the range of 1.27–1.30 g cm^–3^, while the corresponding polymers containing the P=S groups (**P2**, **P4**, and **P6**) showed slightly lower densities of 1.21 g cm^–3^. Despite their slightly lower densities, the polymers containing the P=S groups exhibited higher refractive indices, suggesting that their enhanced refractive indices primarily originate from the high molar refractivity of the P=S group rather than an increased density.

The refractive indices of these polymers were found to correlate well with their heteroatom (S and P) contents (Figure 4a). In this analysis, the weight fractions of the S and P atoms were employed instead of the volume fractions, because the volumes occupied by the S and P atoms within the polymer chains cannot be readily determined. The use of weight fractions is a practical approximation when polymer densities can be regarded as comparable. Although the densities measured for the polymers (**P1**–**P6**) were not identical, their relatively small variation (1.21–1.30 g cm^–3^) supports the applicability of this approach. Compared to the poly­(ether)­s containing the P=O (**P1**, **P3**, and **P7**) and P=S (**P2**, **P4**, and **P8**) groups and the poly­(thioether) containing the P=O groups (**P5**), the poly­(thioether)­s containing the P=S groups (**P6**, **P9**, and **P11**) possess higher contents of the S and P atoms, which may contribute to their higher *n*_D_ values. The *n*_D_ value of **P10** is slightly lower than the expected value based on its content of the S and P atoms, likely owing to the large free volume of the SO_2_ group. On the other hand, there is a negative correlation between the refractive indices and the C=C unit content of the polymers (Figure S49), probably because an increase in the C=C unit content leads to a decrease in the S and P atom content, and the C=C unit has a lower *R*_M_ value than the S and P atoms. These results suggest that the P=S group with a high content of heavy heteroatoms is a favorable functional group for designing high-refractive-index materials.

To discuss the wavelength dependence of the refractive indices of the polymers, the Abbe numbers (*ν*_D_ values) were calculated according to eq 2:^1,4^

2 $$\nu_{D}=\frac{n_{D}-1}{n_{F}-n_{C}}$$

The *ν*_D_ values of **P1**–**P10** ranged from 16.5 to 23.8, decreasing as the refractive indices of the polymers increased (Table 1 and Figure 4b). This trend is consistent with the well-known relationship between refractive indices and the Abbe numbers.^1,4,17^ On the other hand, **P11** exhibited a *ν*_D_ value of 21.2, which is higher than those of the polymers with similar refractive indices (**P5**, **P6**, and **P9**) and slightly deviates from the trend observed for **P1**–**P10**. Given that **P11** has the highest P=S group content, this result may suggest that the P=S group exhibits a smaller wavelength dependence of the refractive index in comparison to the C=C and thioether groups. The relatively low dispersion of the P=S group is also supported by theoretical analysis based on density functional theory calculations for the model compounds (Table S2). Although the Abbe number of **P11** is still relatively low for optical polymeric materials, P=S group-containing aliphatic polymers, which are free of aromatic units with large optical dispersion, are expected to achieve higher Abbe numbers while maintaining high refractive indices. Further studies based on this unique potential of the P=S group will be addressed in our forthcoming paper.

## Conclusions

In this study, we developed aromatic poly­(thioether)­s containing phosphine sulfide (P=S) groups and evaluated their properties in comparison with those of their structural analogs, aromatic poly­(ether)­s containing the P=S and phosphine oxide (P=O) groups, as well as a poly­(thioether) containing the P=O groups. The poly­(thioether)­s containing the P=S groups exhibited excellent thermal stability, with 5% weight loss temperatures above 440 °C and glass transition temperatures above 170 °C, and high optical transparency in the visible region with transmittance above 77% at 400 nm. Furthermore, these polymers achieved higher refractive indices, ranging from 1.725 to 1.757 at 589 nm, than the corresponding polymers containing either the P=S or thioether moieties. The refractive indices of these poly­((thio)­ether)­s containing the P=O or P=S groups positively correlate with the heavy heteroatom (S and P) content of the polymers, indicating that the combination of the P=S groups and the thioether linkages effectively enhances the refractive indices of the polymers. The Abbe numbers of the poly­(thioether)­s containing the P=S groups range from 16.5 to 21.2, which are comparable to or slightly higher than those of the polymers with similar refractive indices. The above results highlight the potential of the P=S group, derived from the presence of multiple bonds between the heavy heteroatoms, for designing a new class of high-refractive-index materials and expanding the structural diversity of polymeric materials for optical applications.

## Supplementary Material



## Notes

Synthetic procedures, SEC traces, NMR spectra (^1^H, ^13^C, and ^31^P), densities of polymers, stability test results, and density functional theory calculation results of model compounds (PDF)
