# Supplementary material for: Synthesis of Aromatic Poly(Thioether)s with Phosphine Sulfide Groups for High-Refractive-Index Materials
Source: ACS Omega. 2026 Apr 1;11(14):22220–7. doi: 10.1021/acsomega.5c13502 (PMC13084494; doi:10.1021/acsomega.5c13502)
Supplement: Supplementary file 1 [file ao5c13502_si_001.pdf]

Supporting Information

Synthesis of Aromatic Poly(thioether)s with Phosphine Sulfide Groups for High-Refractive-Index Materials

Ryoyu Hifumi,* Akiyoshi Takai, Ikuyoshi Tomita*

Department of Chemical Science and Engineering, School of Materials and Chemical Technology, Institute of Science Tokyo, Nagatsuta-cho 4259-G1-9, Midori-ku, Yokohama 226-8501, Japan.

E-mail: hifumi.r.aa@m.titech.ac.jp

Contents

1.	General Information	S-3
2.	Materials	S-4
3.	Synthetic Procedures and Characterization	S-6
4.	Stability Tests	S-43
5.	Optical Properties	S-44
6.	References	S-46

1. General Information

Nuclear magnetic resonance (^{1}H, ^{13}C, and ^{31}P NMR) spectra were recorded using a JEOL ECZ-400S instrument at 400, 101, and 162 MHz, respectively. The samples were analyzed in chloroform-d_1 ($CDCl_3$), and the chemical shift values were expressed relative to tetramethylsilane as an internal standard for the ^{1}H and ^{13}C NMR spectra and relative to 85% H_3PO_4 aq as an external standard for the ^{31}P NMR spectra.

High-resolution mass spectra (HRMS) were obtained using a Bruker micrOTOF II spectrometer.

Melting points were determined in capillary tubes using a Stuart SMP10 melting point apparatus.

Size exclusion chromatography (SEC) measurements were performed on a SHIMADZU LC-10AD VP equipped with a guardcolumn H_{HR}-H, a GMH_{XL} column, and a GMH_{HR}-M column (TOSOH TSKgel) using 1-methyl-2-pyrrolidone (NMP) with lithium bromide (LiBr, 10 mM) as an eluent at a flow rate of 0.6 mL/min at 60 °C or a SHIMADZU LC-9A equipped with a guardcolumn H_{HR}-H, and two GMH_{HR}-M tandem columns (TOSOH TSKgel) using chloroform ($CHCl_3$) as an eluent at a flow rate of 1.0 mL/min at 40 °C, after calibration with poly(styrene) standards.

Thermogravimetric analysis (TGA) measurements were performed using a SHIMADZU TGA-50 instrument at a heating rate of 10 °C/min under nitrogen. Differential scanning calorimetry (DSC) measurements were performed using a SHIMADZU DSC-60 instrument at a heating rate of 20 °C/min under nitrogen.

Ultraviolet–visible (UV–vis) absorption spectra in $CHCl_3$ solutions and transmittance spectra of films were recorded using a Shimadzu UV-3100PC spectrometer. Refractive indices were evaluated using an Abbe refractometer (ATAGO DR-M4) at wavelengths of 486, 589, and 656 nm.

The density functional theory calculations with the Becke-three-parameter-Lee–Yang–Parr hybrid (B3LYP) were performed using the Gaussian 16 (Revision C.01) program package.[1] The 6-31G(d,p) basis set was used for the geometry optimizations and the 6-311+G(2d,p) basis set was used for the calculations of frequency-dependent polarizabilities at a wavelength of 589.3 nm.

2. Materials

Bis(4-hydroxyphenyl) ether, bis(4-hydroxyphenyl) sulfide, 1,3-dimercaptobenzene, and Lawesson's reagent were purchased from Tokyo Chemical Industry and used as received. Bis(4-mercaptophenyl) sulfide and lithium bromide (LiBr) were purchased from Sigma-Aldrich and used as received. Bis(4-fluorophenyl) sulfone was purchased from Angene Chemical and used as received. Resorcinol was purchased from Sumitomo Chemical and used as received. Sodium hydroxide (NaOH) was purchased from Kanto Chemical and used as received. Sodium hydrosulfide n-hydrate ($NaSH{\bullet}nH_2O$, 65wt%), potassium carbonate (K_2CO_3), and hydrochloric acid (HCl aq, 35%) were purchased from FUJIFILM Wako Pure Chemical and used as received.

N,*N*-Dimethylformamide was purchased from Godo and distilled over CaH_2 under vacuum prior to use. 1-Methyl-2-pyrrolidone (NMP) was purchased from Godo and distilled over CaH_2

under vacuum prior to use in synthesis or was used as received for the SEC measurements. Acetone, methanol, tetrahydrofuran (THF), and toluene were purchased from Godo and used as received. Chloroform ($CHCl_3$) was purchased from Shin-Etsu Chemical and used as received. Chloroform-d_1 ($CDCl_3$) was purchased from Kanto Chemical and used as received.

Silica gel (SiO_2) for column chromatography was purchased from Kanto Chemical (Silica gel 60N, spherical, neutral, particle size 63–210 μm) and used as received.

3. Synthetic Procedures and Characterization

Bis(4-mercaptophenyl)phenylphosphine sulfide

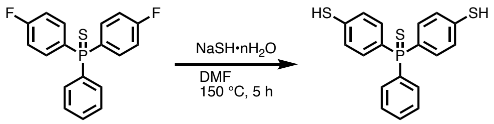

Scheme S1. Synthesis of bis(4-mercaptophenyl)phenylphosphine sulfide.

The synthetic procedure is described in the main text. The ^{1}H, ^{13}C, and ^{31}P NMR spectra are shown below.

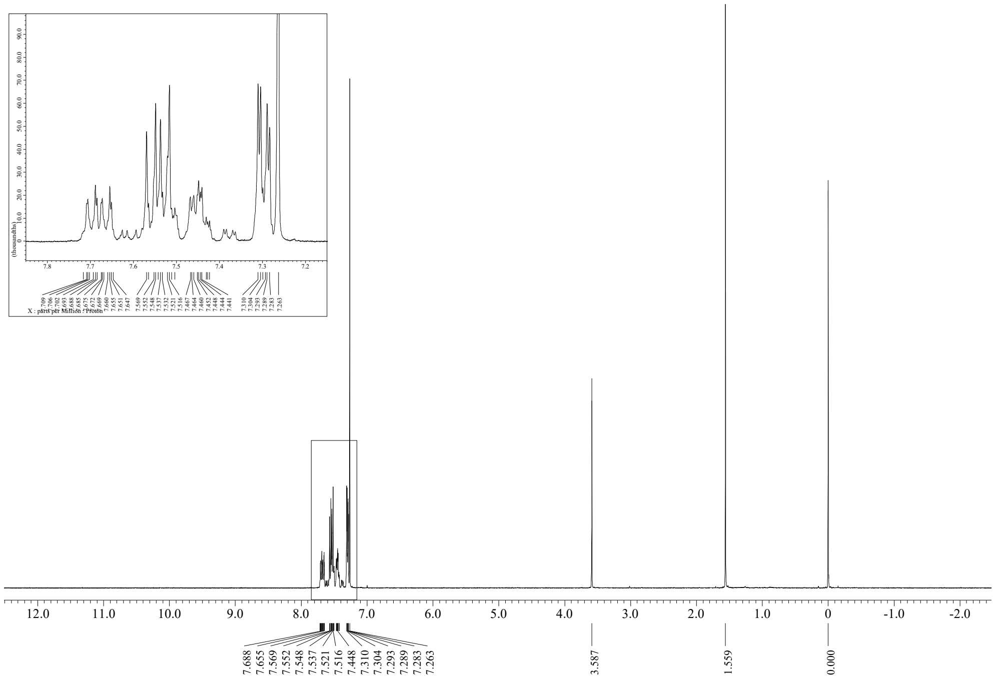

Figure S1. ^{1}H NMR spectrum of bis(4-mercaptophenyl)phenylphosphine sulfide in $CDCl_3$.

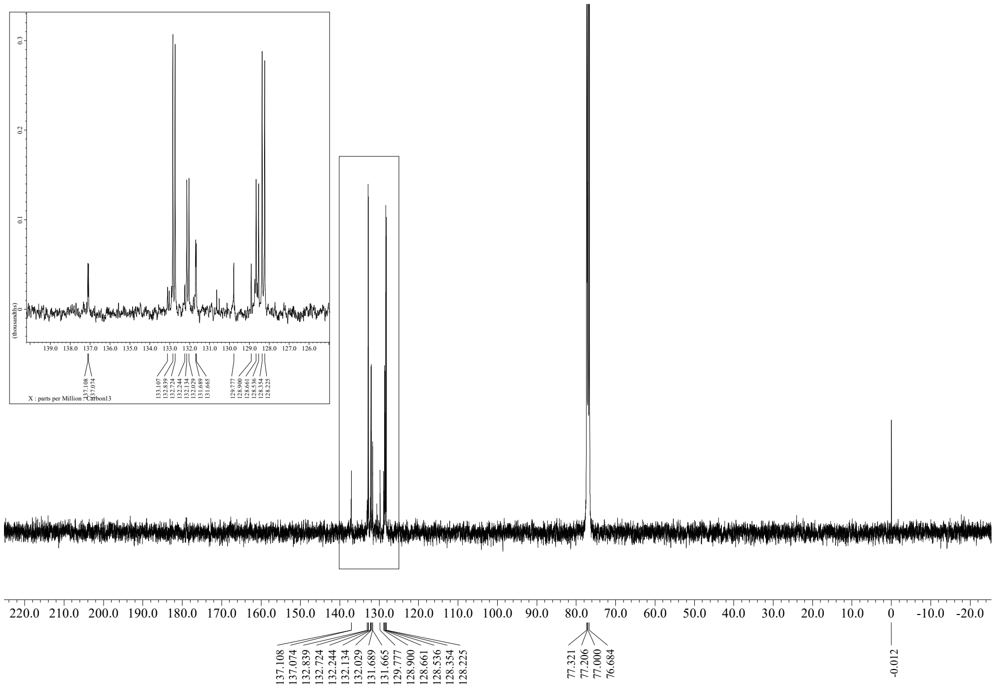

Figure S2. ^{13}C NMR spectrum of bis(4-mercaptophenyl)phenylphosphine sulfide in $CDCl_3$.

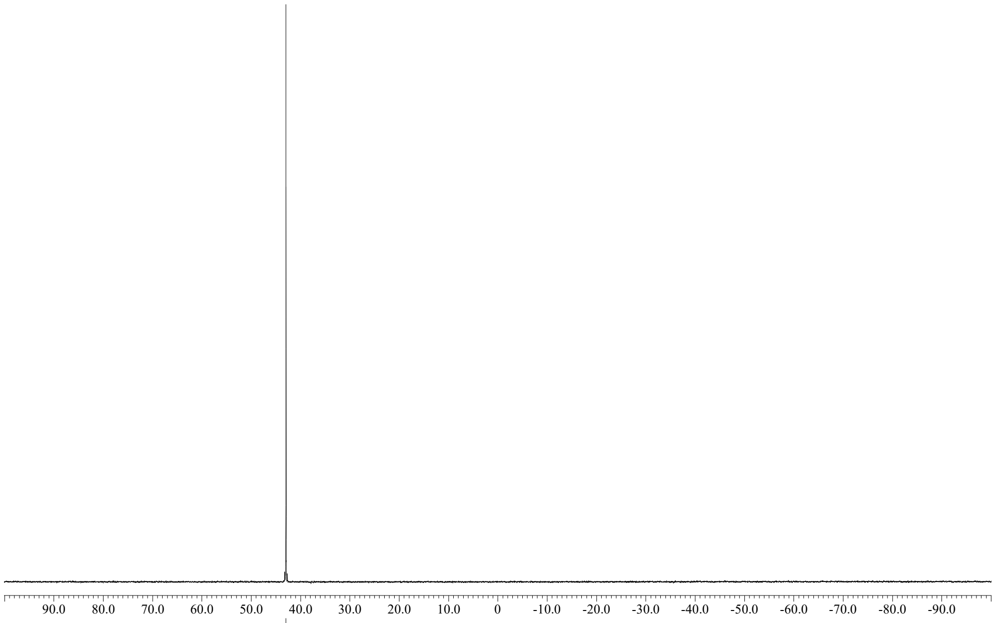

Figure S3. ^{31}P NMR spectrum of bis(4-mercaptophenyl)phenylphosphine sulfide in $CDCl_3$.

Typical procedure for polymerization (Procedure A)

The polymerization was performed according to the procedure described in our previous publications.[2,3]

An arylene difluoride monomer (1 eq.) and a bis(thio)phenol monomer (1 eq.) were reacted in the presence of K_2CO_3 (1.4–2.2 eq.) in NMP at 130 °C for 1–2.5 h and then at 180 °C for several hours under nitrogen or argon. After cooling to ambient temperature, the reaction mixture was diluted with THF and filtered through Kiriyama No. 5B filter paper to remove insoluble salts. The filtrate was poured into dilute HCl aq, and the resulting solid was washed with deionized water (twice) and acetone (3 times). After dissolving the obtained solid in THF, the solution was poured into deionized water, and the resulting solid was washed with deionized water (3 times) and dried in a vacuum oven at 120 °C for several hours.

Typical procedure for converting phosphine oxide to phosphine sulfide groups (Procedure B)

The reactions were performed according to the procedure described in our previous publications.[2]

A poly((thio)ether) with phosphine oxide groups (1 eq.) and Lawesson's reagent (ca. 0.85 eq.) were reacted in toluene under reflux for 1–2 d under nitrogen or argon. The reaction mixture was cooled to ambient temperature, and the volatile fraction was evaporated under reduced pressure. After the residue was dissolved in $CHCl_3$, the solution was poured into acetone, and the resulting solid was washed with acetone (twice). The solid was dissolved in THF, and the resulting

solution was poured into methanol. The resulting solid was washed with methanol (3 times) and dried in a vacuum oven at 120 °C for several hours.

P1

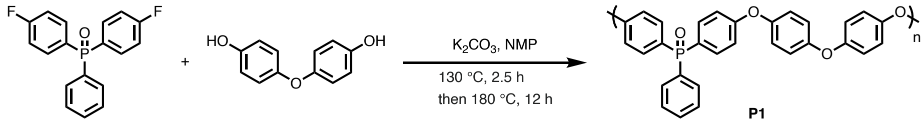

Scheme S2. Synthesis of **P1**.

The synthesis of **P1** was described in our previous paper.[4]

According to the procedure A, bis(4-fluorophenyl)phenylphosphine oxide (4.09 g, 13.0 mmol, 1 eq.) and bis(4-hydroxyphenyl) ether (2.60 g, 12.9 mmol, 0.992 eq.) were reacted in the presence of K_2CO_3 (2.84 g, 20.5 mmol, 1.58 eq.) in NMP (22 mL) at 130 °C for 2.5 h and then at 180 °C for 12 h to give the polymer (**P1**). The yield was 5.68 g (92.0% yield).

M_n = 25900, M_w = 77800 (SEC in NMP with LiBr).

^{1}H NMR (400 MHz, $CDCl_3$) δ 7.69–7.52 (7H), 7.48–7.43 (2H), 7.06–7.00 (12H) ppm.

^{13}C{^{1}H} NMR (101 MHz, $CDCl_3$) δ 161.3 (d, $^4J_{C–P}$ = 2.9 Hz), 153.9, 150.8, 134.1 (d, $J_{C–P}$ = 11.6 Hz), 132.8 (d, $^1J_{C–P}$ = 105.5 Hz), 132.0, 131.9, 128.5 (d, $J_{C–P}$ = 12.0 Hz), 126.0 (d, $^1J_{C–P}$ = 108.9 Hz), 121.7, 120.1, 117.1 (d, $J_{C–P}$ = 13.0 Hz) ppm.

^{31}P{^{1}H} NMR (162 MHz, $CDCl_3$) δ 29.0 (s) ppm.

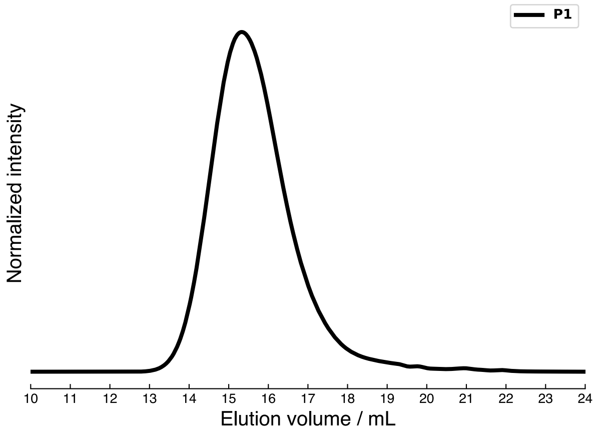

Figure S4. SEC traces of **P1** in NMP with LiBr (10 mM).

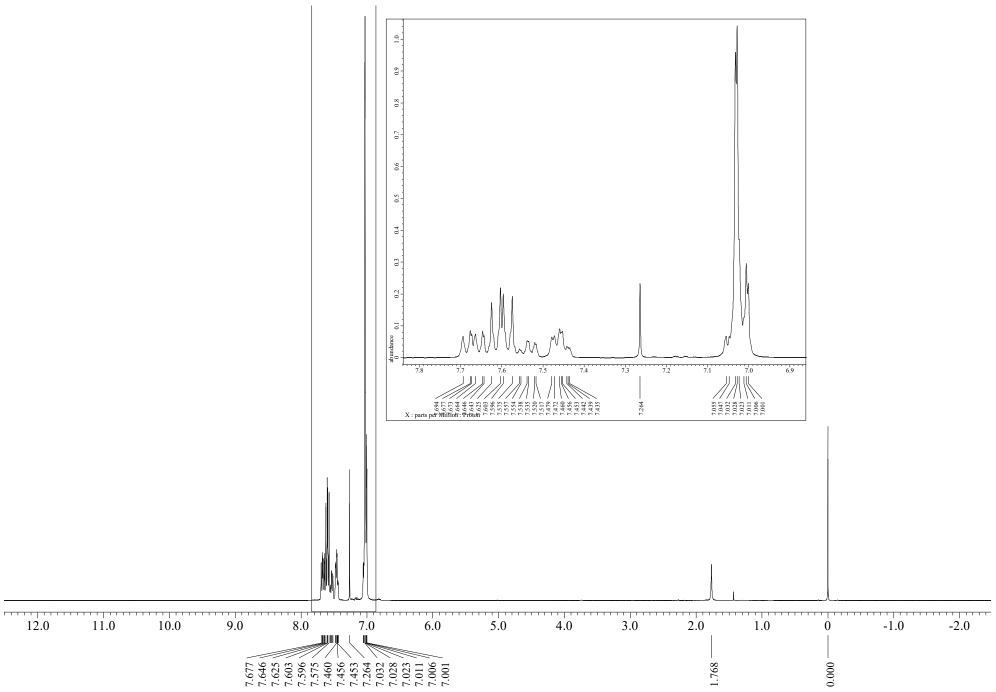

Figure S5. ^{1}H NMR spectrum of **P1** in $CDCl_3$.

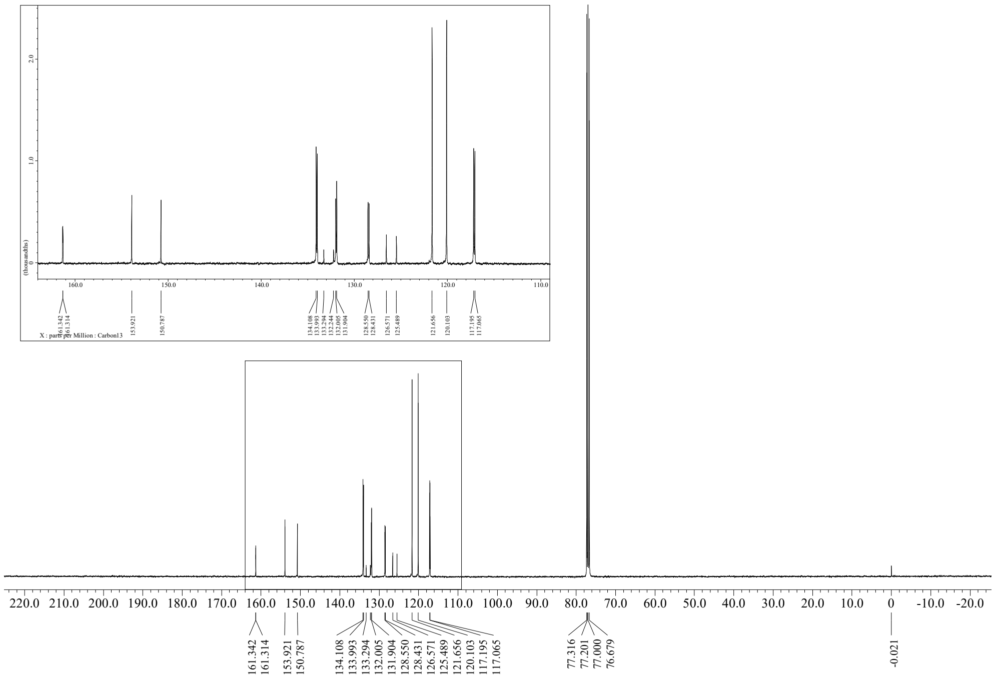

Figure S6. ^{13}C NMR spectrum of **P1** in $CDCl_3$.

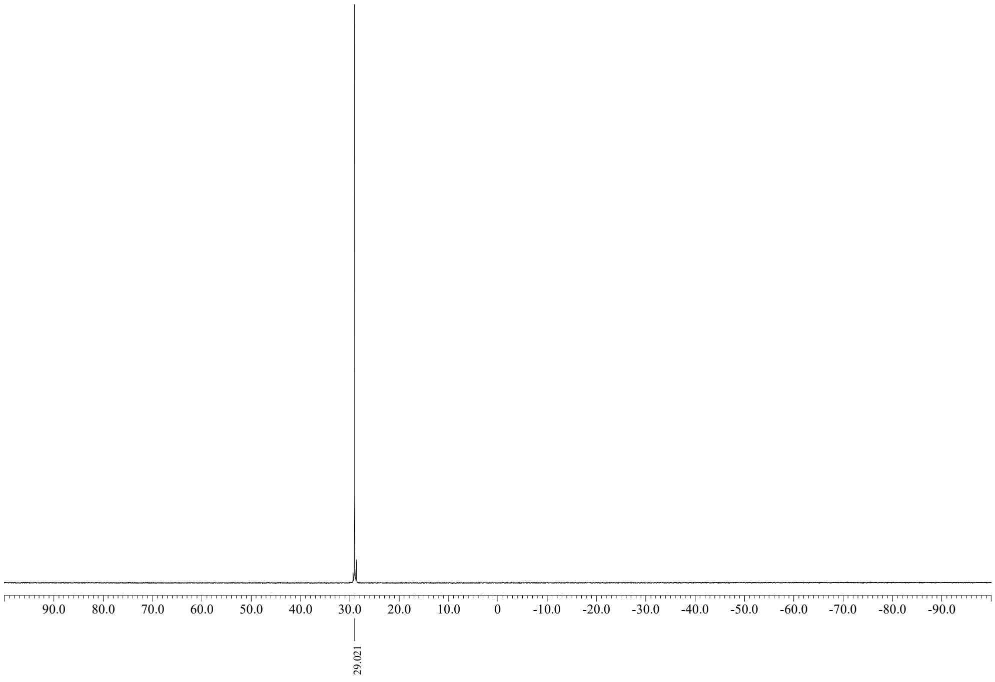

Figure S7. ^{31}P NMR spectrum of **P1** in $CDCl_3$.

P2

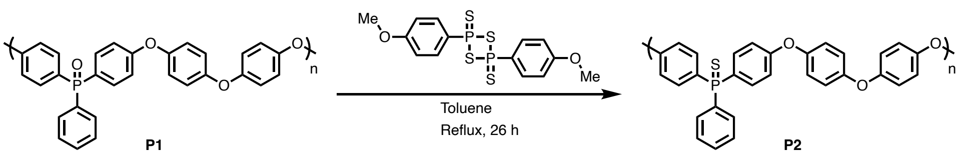

Scheme S3. Synthesis of **P2**.

The synthesis of **P2** was described in our previous paper.[4]

According to the procedure B, **P1** (3.09 g, 6.49 mmol, 1 eq.) and Lawesson's reagent (2.23 g, 5.51 mmol, 0.849 eq.) were reacted in toluene (80 mL) under reflux for 26 h to give the polymer (**P2**). The yield was 2.89 g (90.4% yield).

M_n = 43300, M_w = 88500 (SEC in NMP with LiBr).

^{1}H NMR (400 MHz, $CDCl_3$) δ 7.74–7.61 (6H), 7.52–7.41 (3H), 7.05–6.98 (12H) ppm.

^{13}C{^{1}H} NMR (101 MHz, $CDCl_3$) δ 161.1 (d, $^4J_{C-P}$ = 2.9 Hz), 153.9, 150.8, 134.2 (d, J_{C-P} = 12.5 Hz), 133.2 (d, $^1J_{C-P}$ = 86.2 Hz), 132.1 (d, J_{C-P} = 11.1 Hz), 131.5 (d, $^4J_{C-P}$ = 2.4 Hz), 128.5 (d, J_{C-P} = 13.0 Hz), 126.2 (d, $^1J_{C-P}$ = 90.1 Hz), 121.7, 120.1, 117.1 (d, J_{C-P} = 13.5 Hz) ppm.

^{31}P{^{1}H} NMR (162 MHz, $CDCl_3$) δ 42.4 (s) ppm.

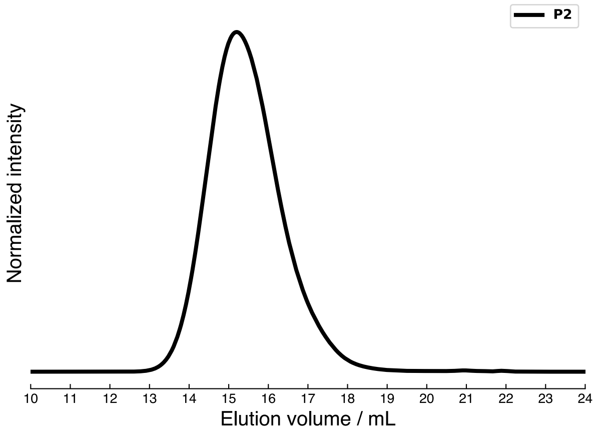

Figure S8. SEC traces of **P2** in NMP with LiBr (10 mM).

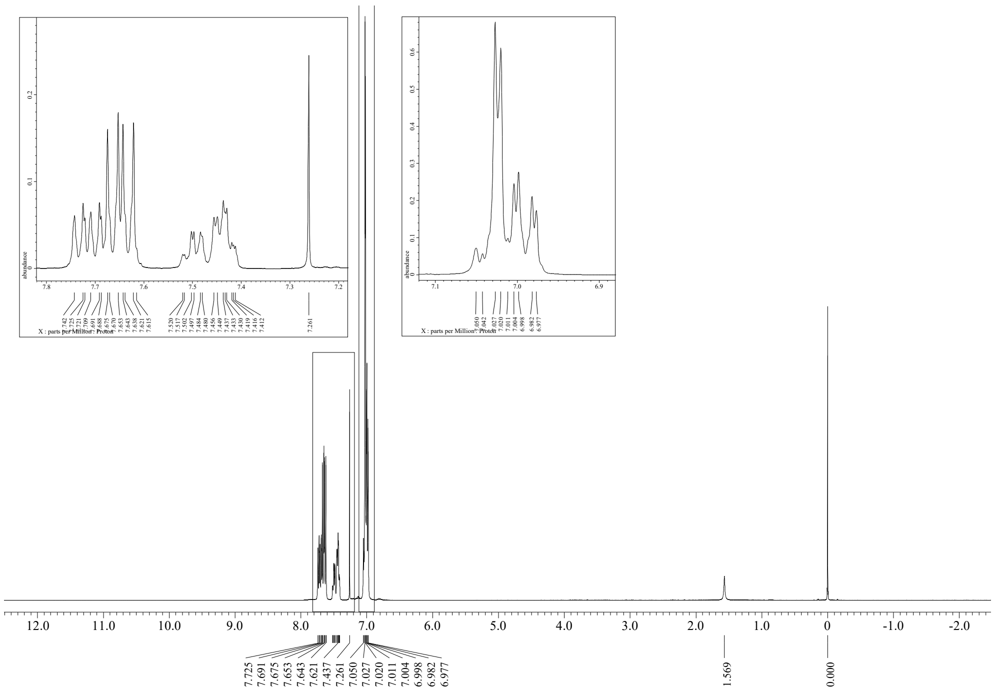

Figure S9. ^{1}H NMR spectrum of **P2** in $CDCl_3$.

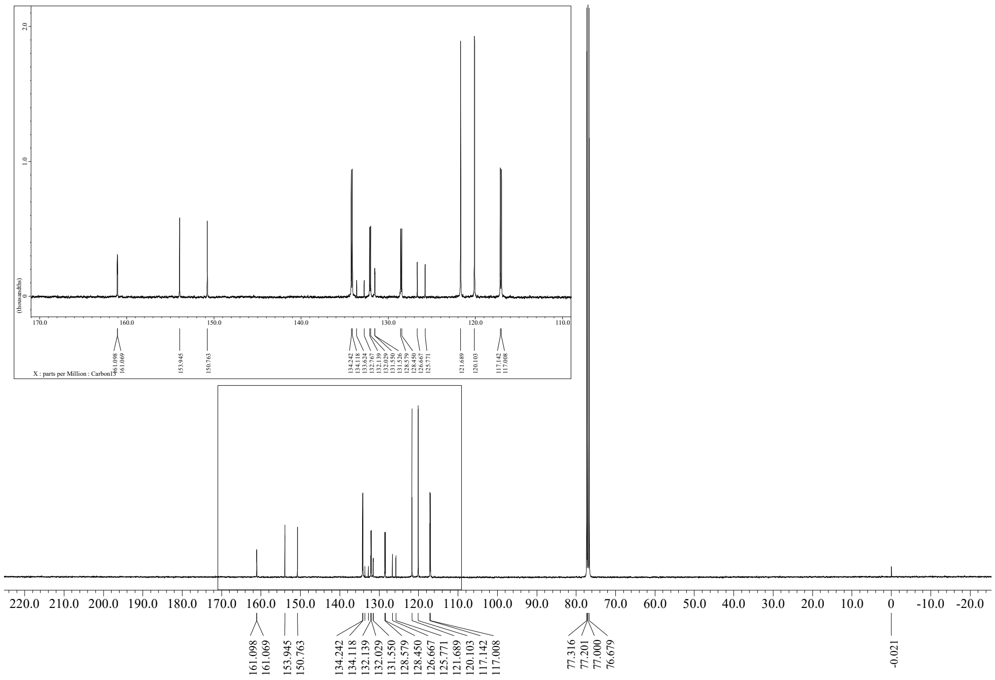

Figure S10. ^{13}C NMR spectrum of **P2** in $CDCl_3$.

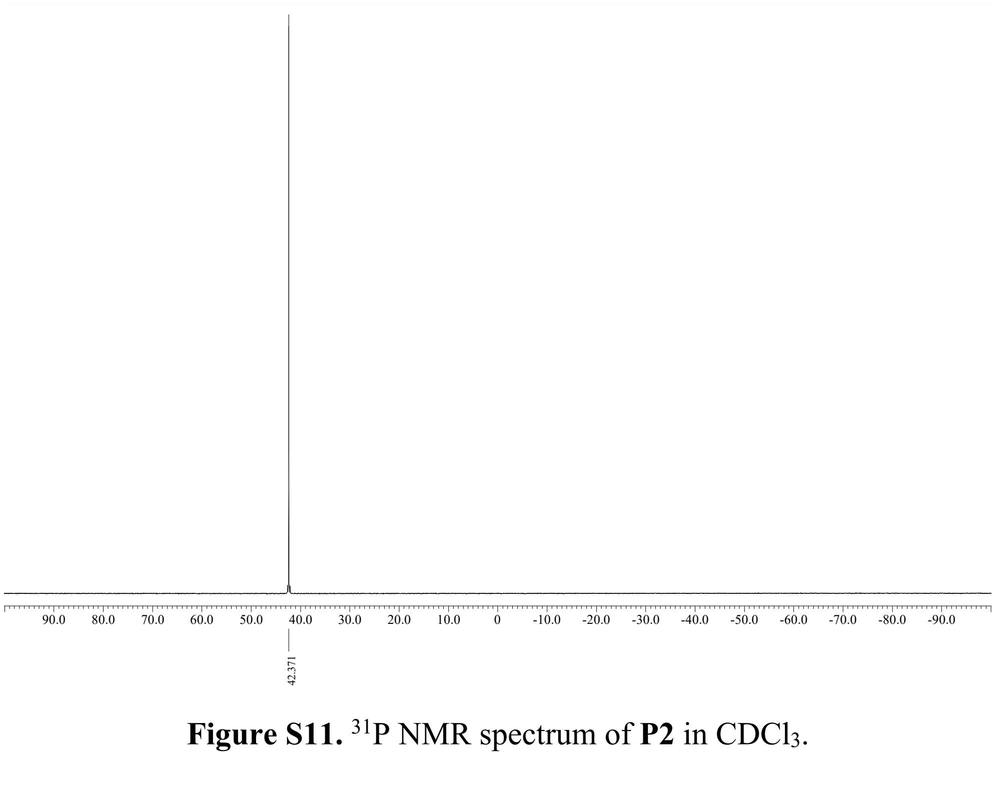

Figure S11. ^{31}P NMR spectrum of **P2** in $CDCl_3$.

P3

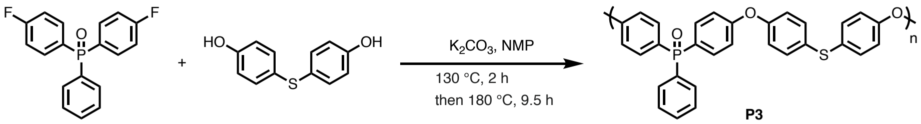

Scheme S4. Synthesis of **P3**.

The synthesis of **P3** was described in our previous paper.[4]

According to the procedure A, bis(4-fluorophenyl)phenylphosphine oxide (3.77 g, 12.0 mmol, 1 eq.) and bis(4-hydroxyphenyl) sulfide (2.57 g, 11.8 mmol, 0.983 eq.) were reacted in the presence of K_2CO_3 (2.77 g, 20.0 mmol, 1.67 eq.) in NMP (20 mL) at 130 °C for 2 h and then at 180 °C for 9.5 h to give the polymer (**P3**). The yield was 5.11 g (87.1% yield).

M_n = 19100, M_w = 106300 (SEC in NMP with LiBr).

^{1}H NMR (400 MHz, $CDCl_3$) δ 7.69–7.52 (7H), 7.47–7.44 (2H), 7.34 (d, J = 8.4 Hz, 4H), 7.05–6.98 (8H) ppm.

^{13}C{^{1}H} NMR (101 MHz, $CDCl_3$) δ 160.4 (d, $^4J_{C–P}$ = 2.9 Hz), 155.0, 134.1 (d, $J_{C–P}$ = 11.1 Hz), 132.8, 132.6 (d, $^1J_{C–P}$ = 105.5 Hz), 132.0, 131.9, 131.2, 128.5 (d, $J_{C–P}$ = 12.0 Hz), 126.7 (d, $^1J_{C–P}$ = 108.4 Hz), 120.7, 117.9 (d, $J_{C–P}$ = 13.5 Hz) ppm.

^{31}P{^{1}H} NMR (162 MHz, $CDCl_3$) δ 28.9 (s) ppm.

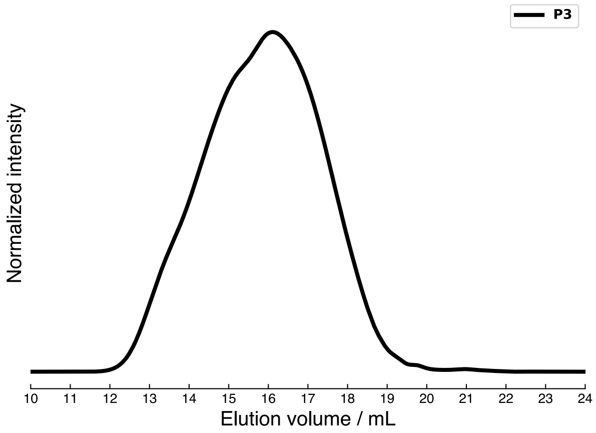

Figure S12. SEC traces of **P3** in NMP with LiBr (10 mM).

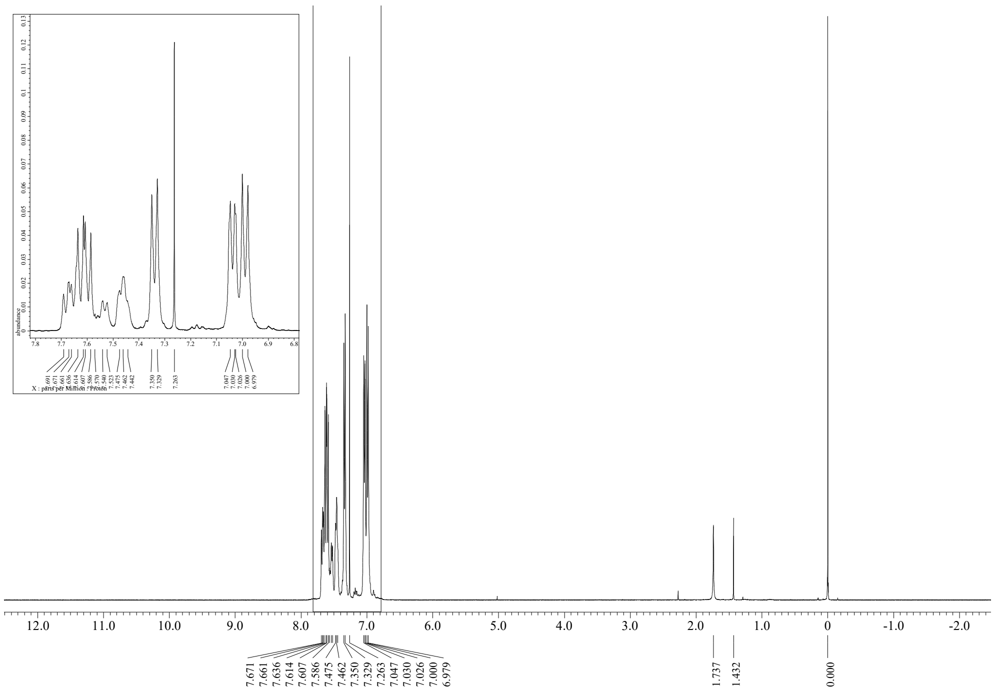

Figure S13. ^{1}H NMR spectrum of **P3** in $CDCl_3$.

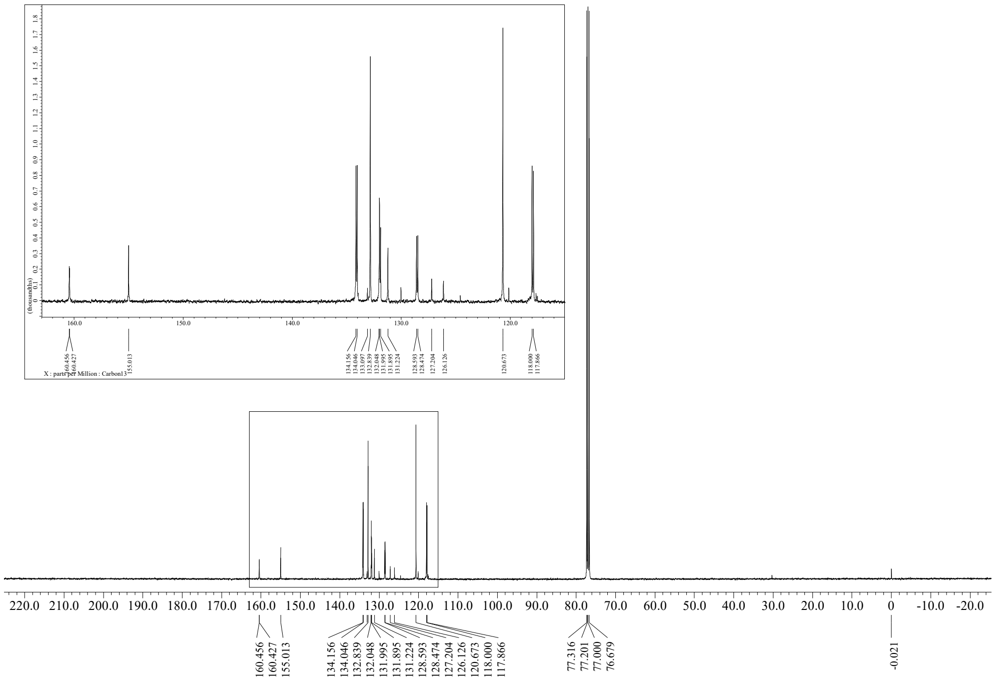

Figure S14. ^{13}C NMR spectrum of **P3** in $CDCl_3$.

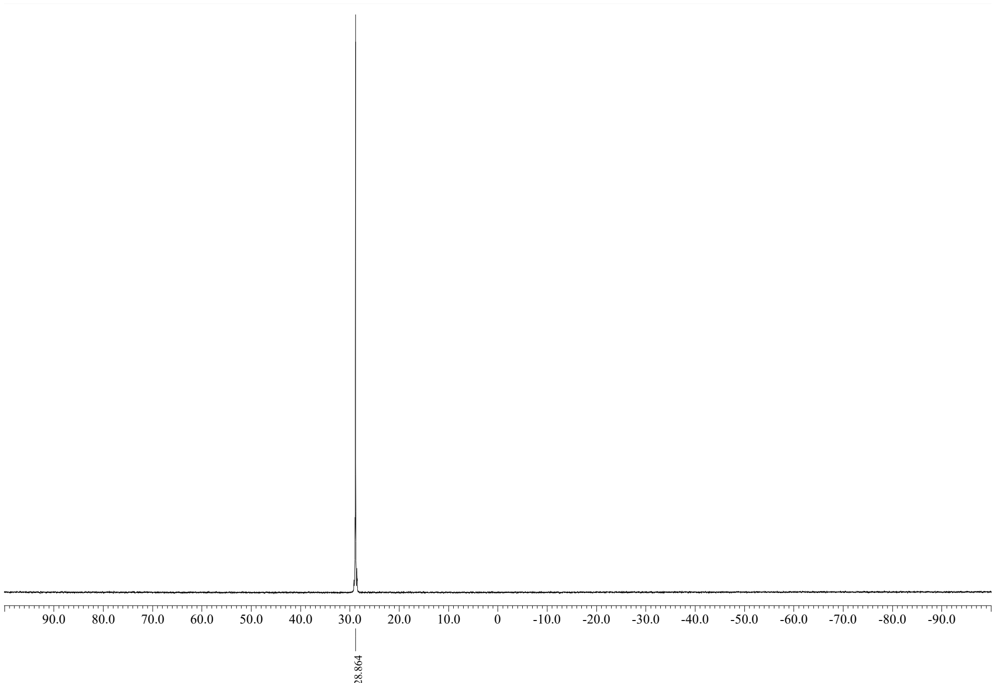

Figure S15. ^{31}P NMR spectrum of **P3** in $CDCl_3$.

P4

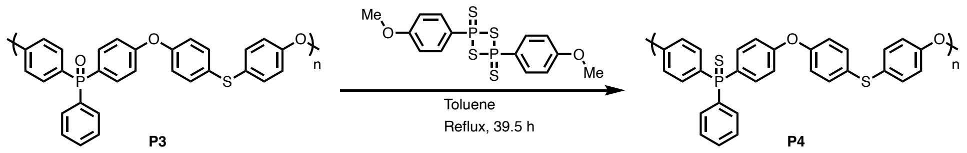

Scheme S5. Synthesis of **P4**.

The synthesis of **P4** was described in our previous paper.[4]

According to the procedure B, **P3** (2.69 g, 5.46 mmol, 1 eq.) and Lawesson's reagent (1.90 g, 4.70 mmol, 0.861 eq.) were reacted in toluene (80 mL) under reflux for 39.5 h to give the polymer (**P4**). The yield was 1.98 g (71.3% yield).

M_n = 27800, M_w = 174300 (SEC in NMP with LiBr).

^{1}H NMR (400 MHz, $CDCl_3$) δ 7.74–7.63 (6H), 7.52–7.42 (3H), 7.33 (d, J = 8.4 Hz, 4H), 7.06–6.94 (8H) ppm.

^{13}C{^{1}H} NMR (101 MHz, $CDCl_3$) δ 160.2 (d, $^4J_{C–P}$ = 2.9 Hz), 155.0, 134.2 (d, $J_{C–P}$ = 12.5 Hz), 133.0 (d, $^1J_{C–P}$ = 85.7 Hz), 132.8, 132.1 (d, $J_{C–P}$ = 11.1 Hz), 131.6 (d, $^4J_{C–P}$ = 2.9 Hz), 131.3, 128.6 (d, $J_{C–P}$ = 13.0 Hz), 126.9 (d, $^1J_{C–P}$ = 89.6 Hz), 120.7, 117.9 (d, $J_{C–P}$ = 13.5 Hz) ppm.

^{31}P{^{1}H} NMR (162 MHz, $CDCl_3$) δ 42.4 (s) ppm.

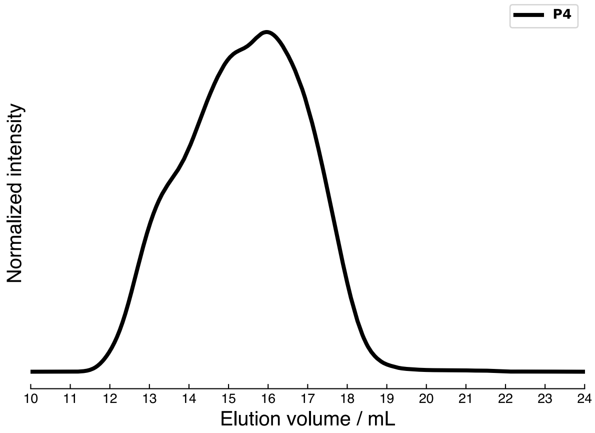

Figure S16. SEC traces of **P4** in NMP with LiBr (10 mM).

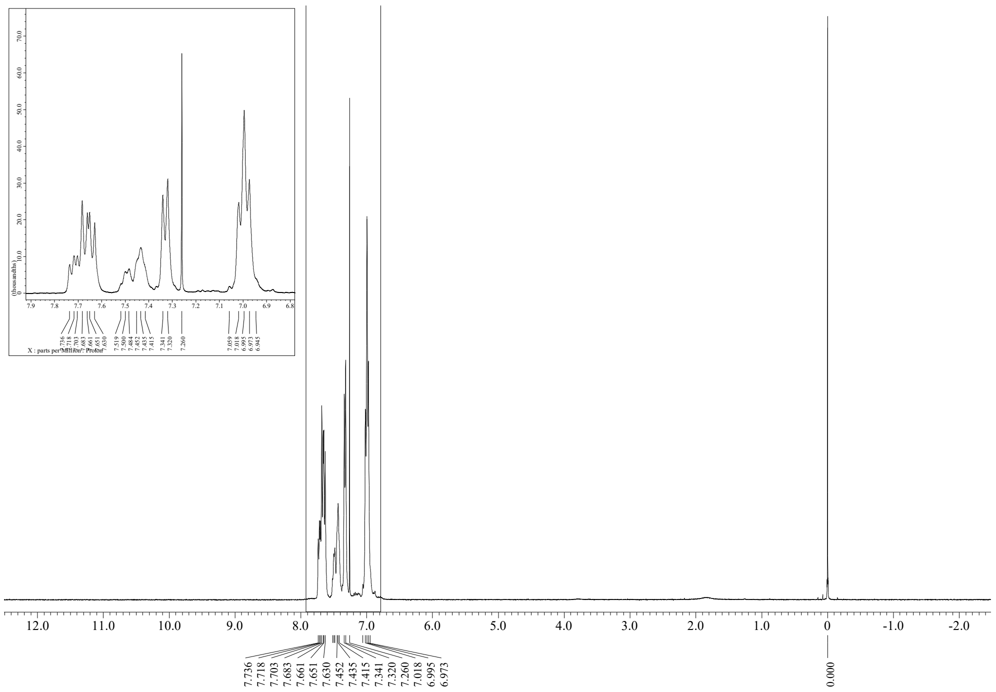

Figure S17. ^{1}H NMR spectrum of **P4** in $CDCl_3$.

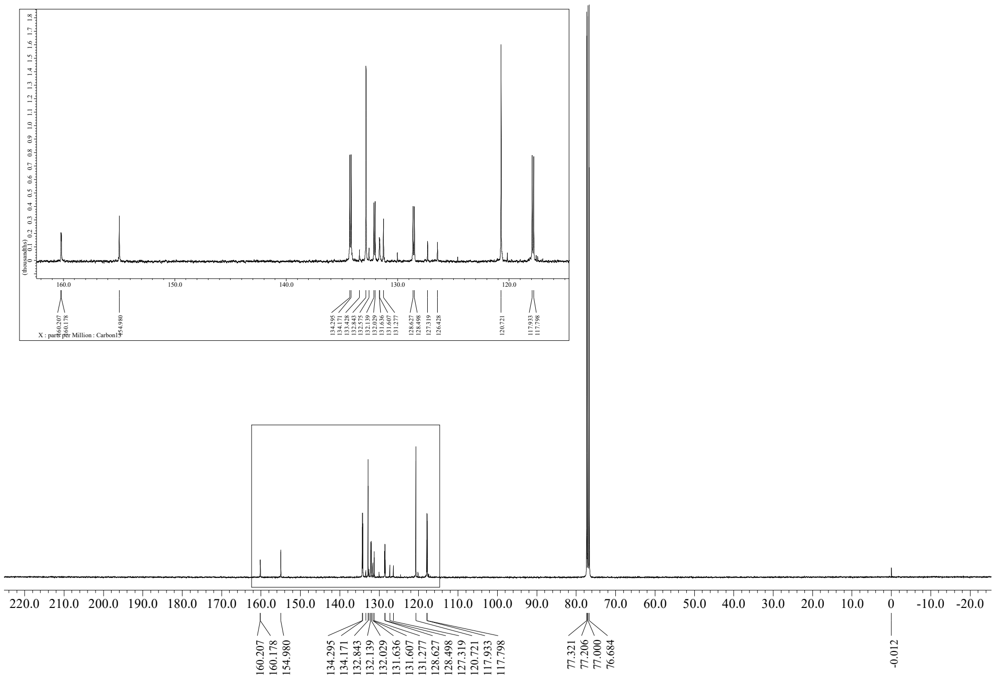

Figure S18. ^{13}C NMR spectrum of **P4** in $CDCl_3$.

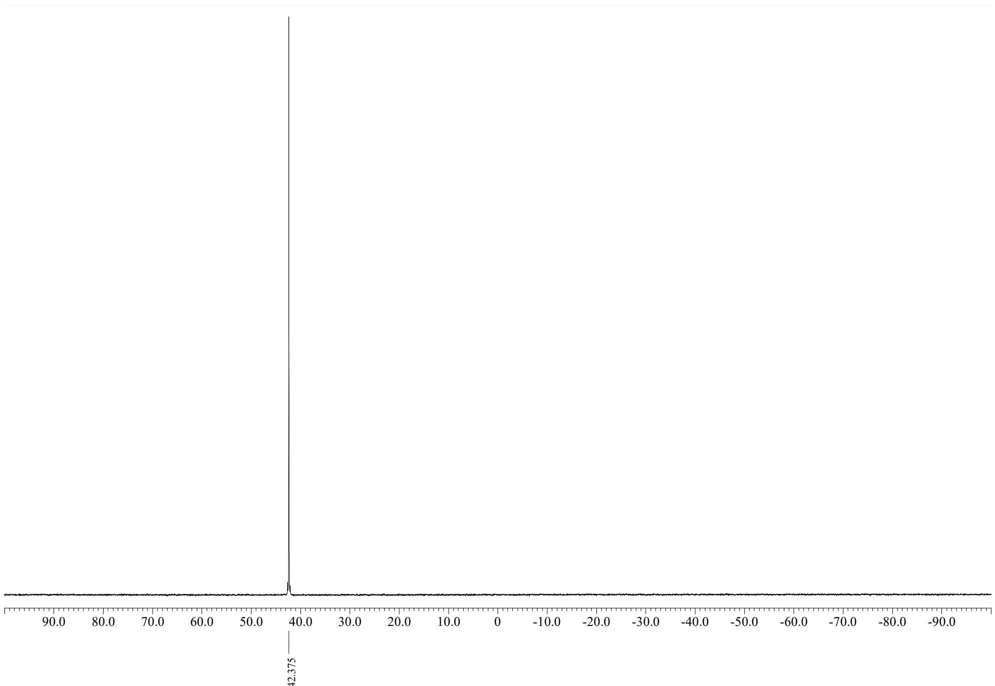

Figure S19. ^{31}P NMR spectrum of **P4** in $CDCl_3$.

P5

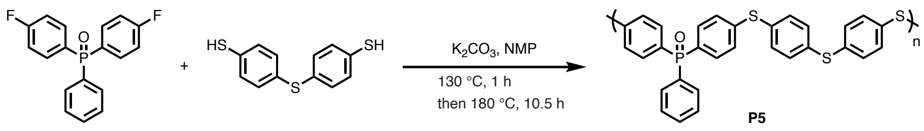

Scheme S6. Synthesis of **P5**.

The synthesis of **P5** was described in our previous paper.[4]

According to the procedure A, bis(4-fluorophenyl)phenylphosphine oxide (3.77 g, 12.0 mmol, 1 eq.) and bis(4-mercaptophenyl) sulfide (2.94 g, 11.7 mmol, 0.975 eq.) were reacted in the presence of K_2CO_3 (2.74 g, 19.8 mmol, 1.65 eq.) in NMP (20 mL) at 130 °C for 1 h and then at 180 °C for 10.5 h to give the polymer (**P5**). The yield was 6.00 g (96.1% yield).

M_n = 22000, M_w = 54700 (SEC in NMP with LiBr).

^{1}H NMR (400 MHz, $CDCl_3$) δ 7.65–7.60 (2H), 7.55–7.48 (5H), 7.46–7.42 (2H), 7.38–7.29 (8H), 7.26–7.23 (4H) ppm.

^{13}C{^{1}H} NMR (101 MHz, $CDCl_3$) δ 142.5 (d, $^4J_{C–P}$ = 2.9 Hz), 135.9, 133.9, 132.6 (d, $J_{C–P}$ = 10.6 Hz), 132.1 (d, $^4J_{C–P}$ = 2.4 Hz), 131.9 (d, $J_{C–P}$ = 10.1 Hz), 131.8, 131.7, 131.5, 129.9 (d, $^1J_{C–P}$ = 106.4 Hz), 128.6 (d, $J_{C–P}$ = 12.0 Hz), 128.2 (d, $J_{C–P}$ = 12.5 Hz) ppm.

^{31}P{^{1}H} NMR (162 MHz, $CDCl_3$) δ 29.0 (s) ppm.

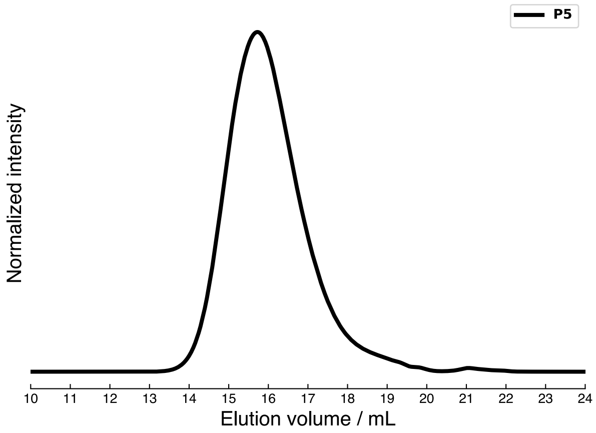

Figure S20. SEC traces of **P5** in NMP with LiBr (10 mM) (Adapted with permission from Hifumi, R.; Tomita, I. Synthesis and Dielectric Properties of Aromatic Poly(thioether)s with Triphenylphosphine Sulfide Moieties. *J. Netw. Polym. Jpn.* **2024**, *45*, 143–150.[4] Copyright 2024 Japan Thermosetting Plastics Industry Association.).

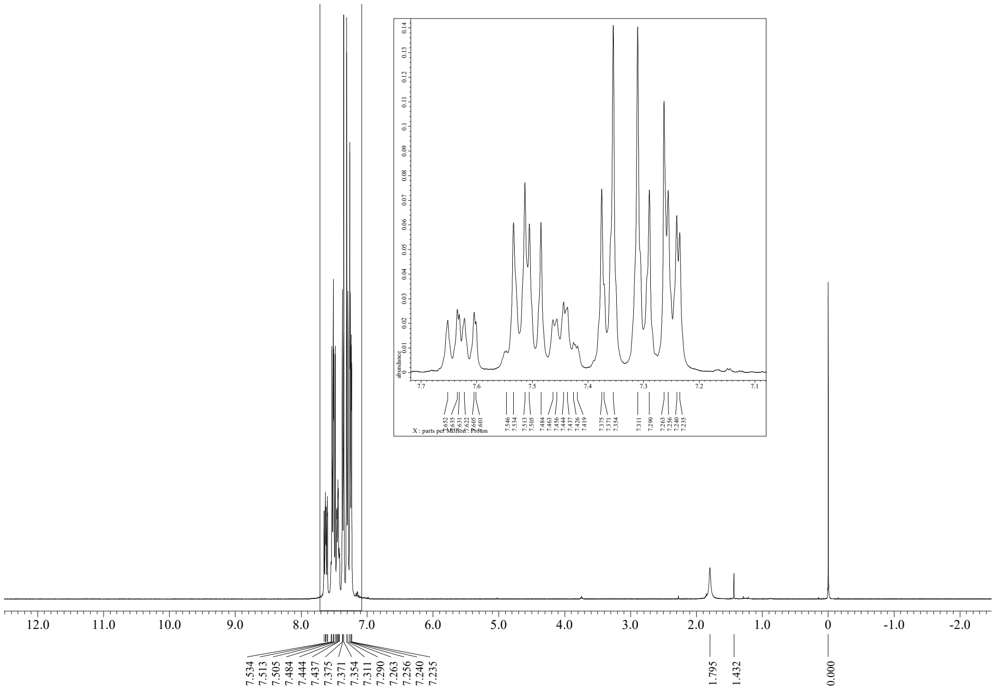

Figure S21. ^{1}H NMR spectrum of **P5** in $CDCl_3$.

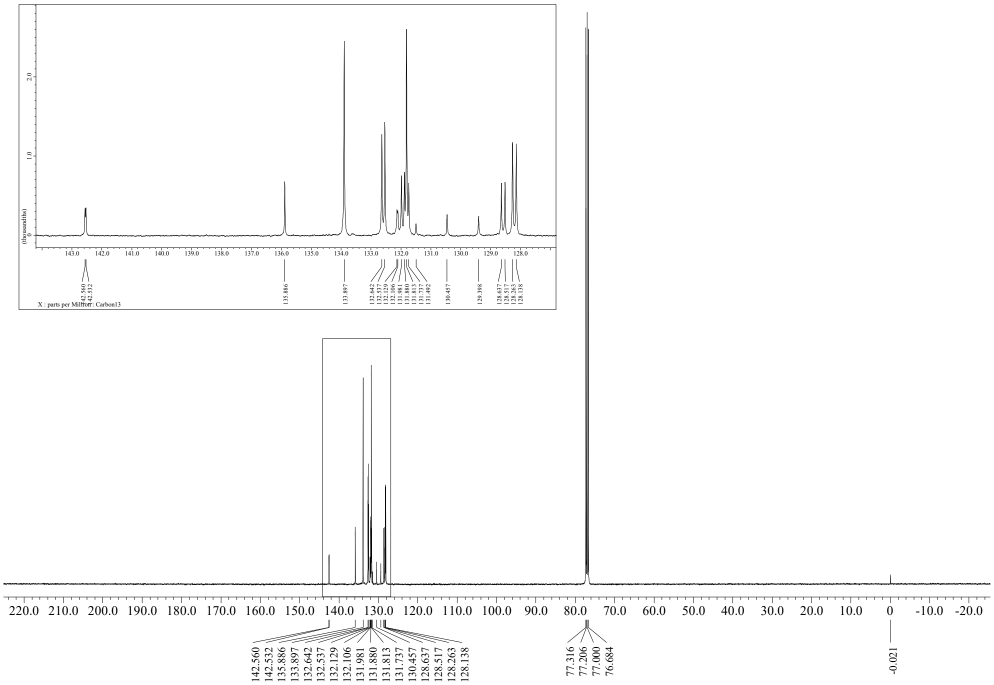

Figure S22. ^{13}C NMR spectrum of **P5** in $CDCl_3$.

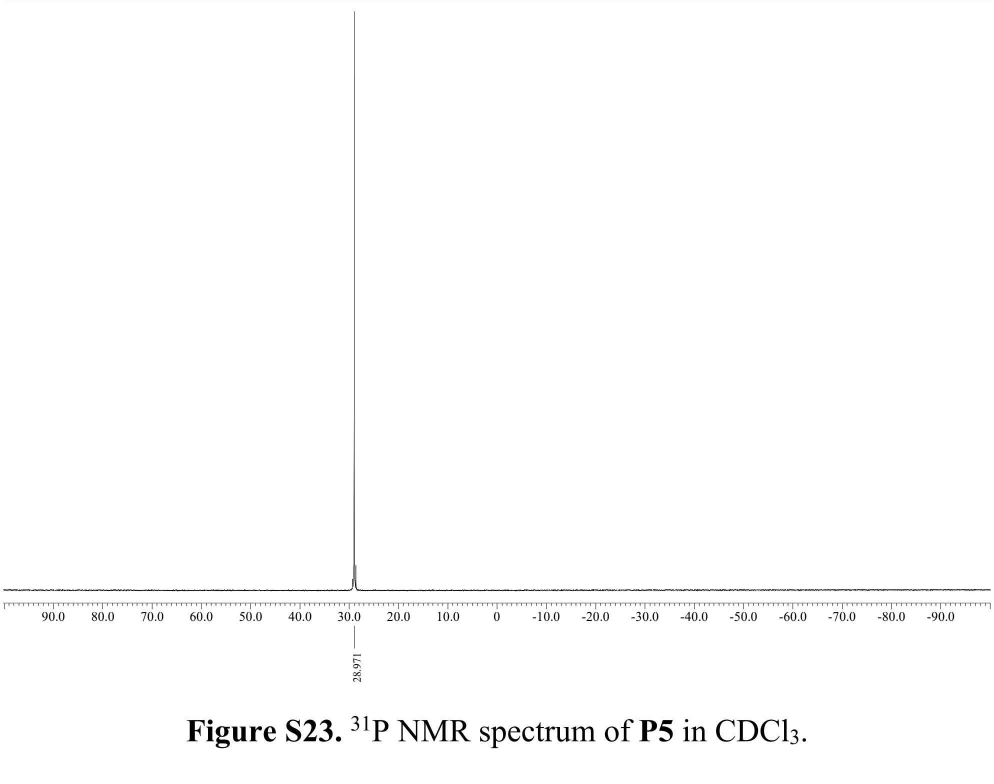

Figure S23. ^{31}P NMR spectrum of **P5** in $CDCl_3$.

P6

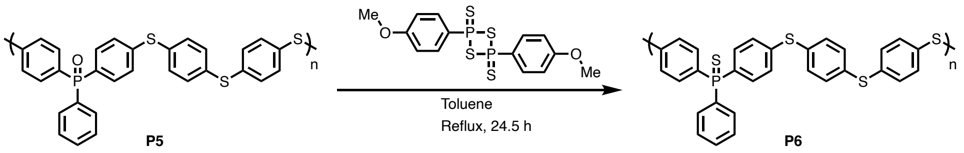

Scheme S7. Synthesis of **P6**.

The synthesis of **P6** was described in our previous paper.[4]

According to the procedure B, **P5** (3.04 g, 5.79 mmol, 1 eq.) and Lawesson's reagent (2.00 g, 4.94 mmol, 0.853 eq.) were reacted in toluene (88 mL) under reflux for 24.5 h to give the polymer (**P6**). The yield was 2.87 g (91.7% yield).

M_n = 28600, M_w = 61900 (SEC in NMP with LiBr).

^{1}H NMR (400 MHz, $CDCl_3$) δ 7.70–7.65 (2H), 7.58–7.47 (5H), 7.44–7.28 (10H), 7.21 (dd, $^{3}J_{H–H}$ = 8.4 Hz, J = 2.3 Hz, 4H) ppm.

^{13}C{^{1}H} NMR (101 MHz, $CDCl_3$) δ 142.3 (d, $^{4}J_{C–P}$ = 3.4 Hz), 136.0, 134.0, 132.7 (d, $J_{C–P}$ = 11.1 Hz), 132.4 (d, $^{1}J_{C–P}$ = 85.7 Hz), 132.1 (d, $J_{C–P}$ = 10.6 Hz), 131.8, 131.7 (d, $^{4}J_{C–P}$ = 2.9 Hz), 131.6, 130.3 (d, $^{1}J_{C–P}$ = 87.2 Hz), 128.6 (d, $J_{C–P}$ = 12.5 Hz), 128.1 (d, $J_{C–P}$ = 13.0 Hz) ppm.

^{31}P{^{1}H} NMR (162 MHz, $CDCl_3$) δ 42.9 (s) ppm.

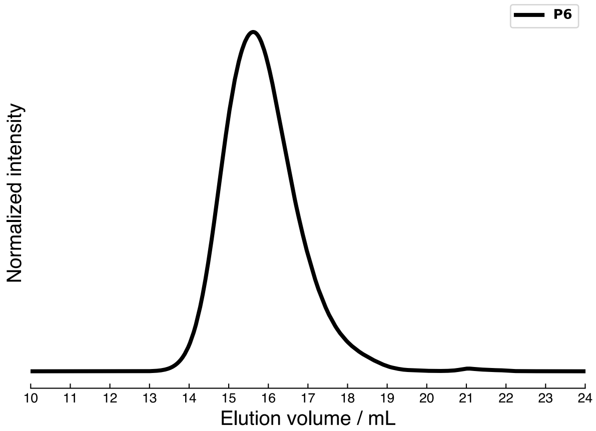

Figure S24. SEC traces of **P6** in NMP with LiBr (10 mM) (Adapted with permission from Hifumi, R.; Tomita, I. Synthesis and Dielectric Properties of Aromatic Poly(thioether)s with Triphenylphosphine Sulfide Moieties. *J. Netw. Polym. Jpn.* **2024**, *45*, 143–150.[4] Copyright 2024 Japan Thermosetting Plastics Industry Association.).

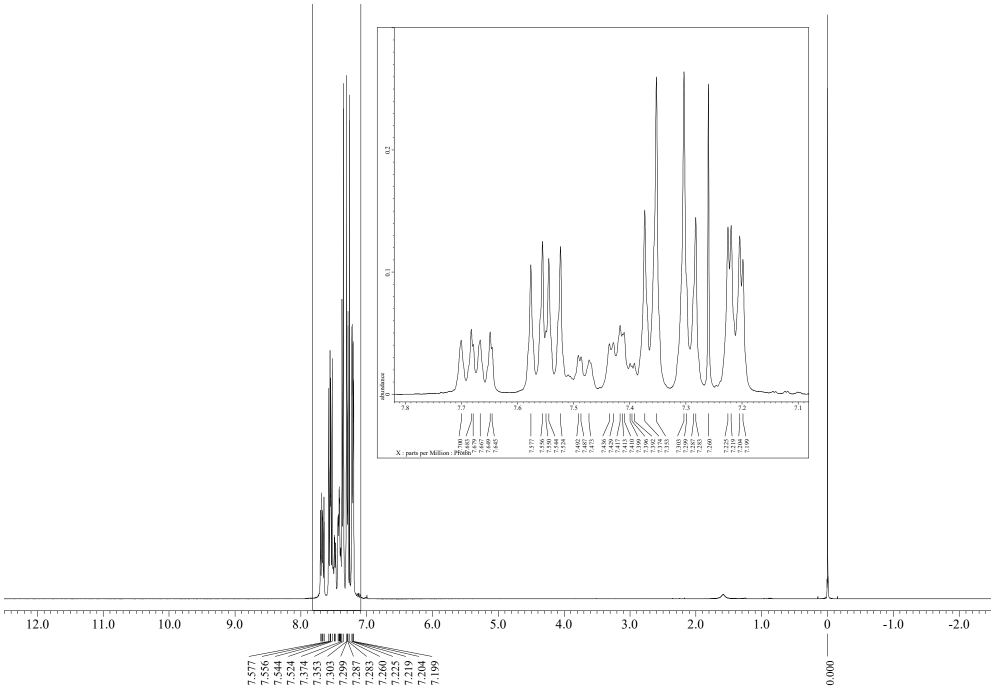

Figure S25. ^{1}H NMR spectrum of **P6** in $CDCl_3$.

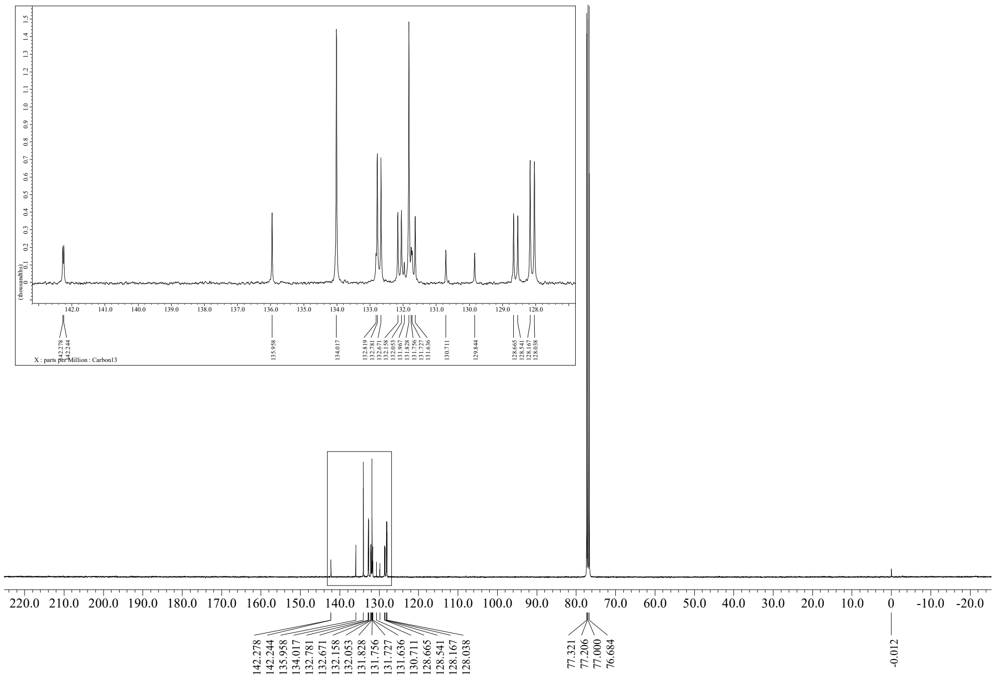

Figure S26. ^{13}C NMR spectrum of **P6** in $CDCl_3$.

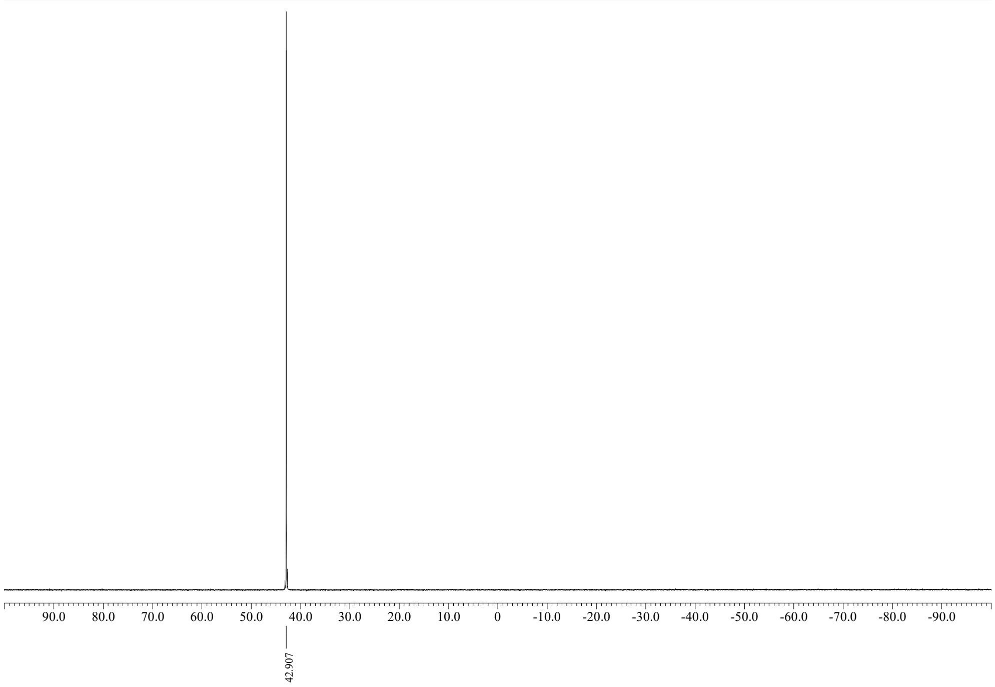

Figure S27. ^{31}P NMR spectrum of **P6** in $CDCl_3$.

P7

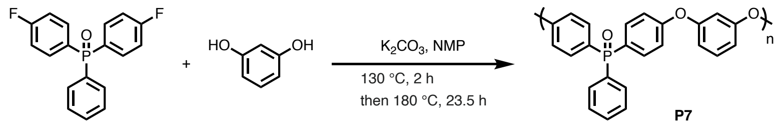

Scheme S8. Synthesis of **P7**.

According to the procedure A, bis(4-fluorophenyl)phenylphosphine oxide (4.95 g, 15.8 mmol, 1 eq.) and resorcinol (1.79 g, 16.3 mmol, 1.03 eq.) were reacted in the presence of K_2CO_3 (3.13 g, 22.6 mmol, 1.43 eq.) in NMP (15 mL) at 130 °C for 2 h and then at 180 °C for 23.5 h to give the polymer (**P7**). The yield was 3.14 g (51.4% yield).

M_n = 12300, M_w = 28400 (SEC in NMP with LiBr).

^{1}H NMR (400 MHz, $CDCl_3$) δ 7.69–7.59 (6H), 7.56–7.52 (1H), 7.48–7.44 (2H), 7.34 (t, $^3J_{H-H}$ = 8.2 Hz, 1H), 7.07–7.04 (4H), 6.84 (dd, $^3J_{H-H}$ = 8.1 Hz, J = 2.1 Hz, 2H), 6.78 (t, $^4J_{H-H}$ = 1.9 Hz, 1H) ppm.

^{13}C{^{1}H} NMR (101 MHz, $CDCl_3$) δ 160.3 (d, $^4J_{C-P}$ = 2.4 Hz), 157.0, 134.1 (d, J_{C-P} = 11.6 Hz), 132.5 (d, $^1J_{C-P}$ = 96.8 Hz), 132.0–131.9 (overlapped), 131.0, 128.6 (d, J_{C-P} = 12.0 Hz), 126.7 (d, $^1J_{C-P}$ = 108.4 Hz), 118.0 (d, J_{C-P} = 13.0 Hz), 115.6, 111.7 ppm.

^{31}P{^{1}H} NMR (162 MHz, $CDCl_3$) δ 29.0 (s) ppm.

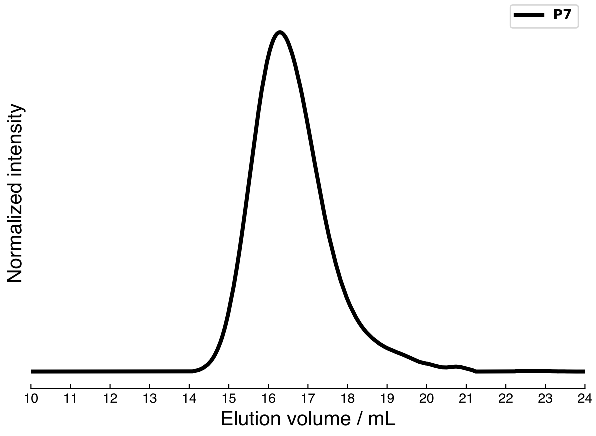

Figure S28. SEC traces of **P7** in NMP with LiBr (10 mM).

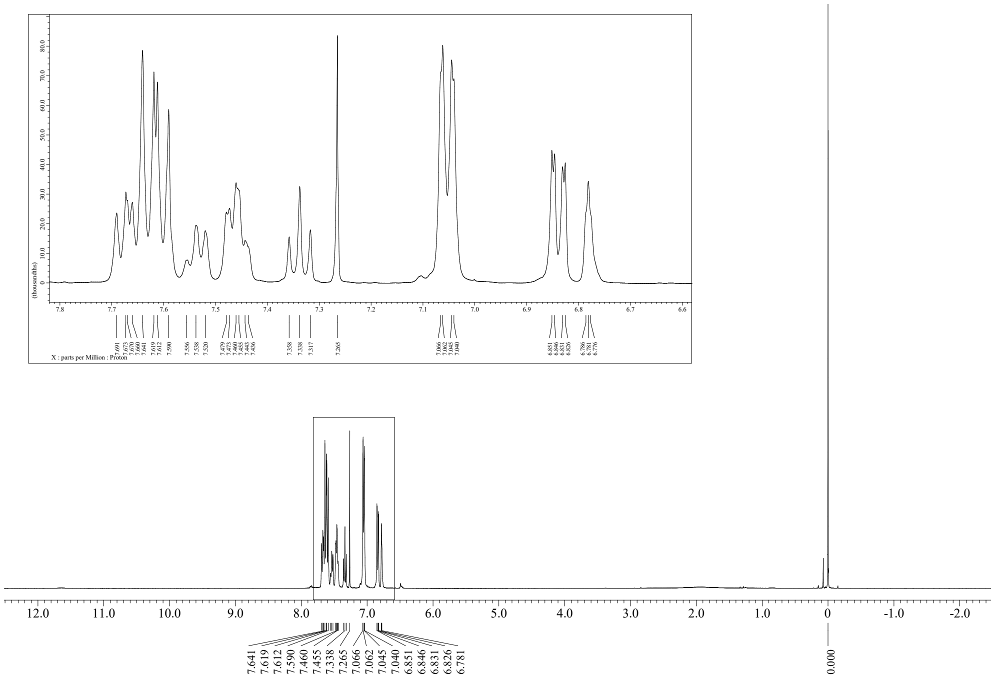

Figure S29. ^{1}H NMR spectrum of **P7** in $CDCl_3$.

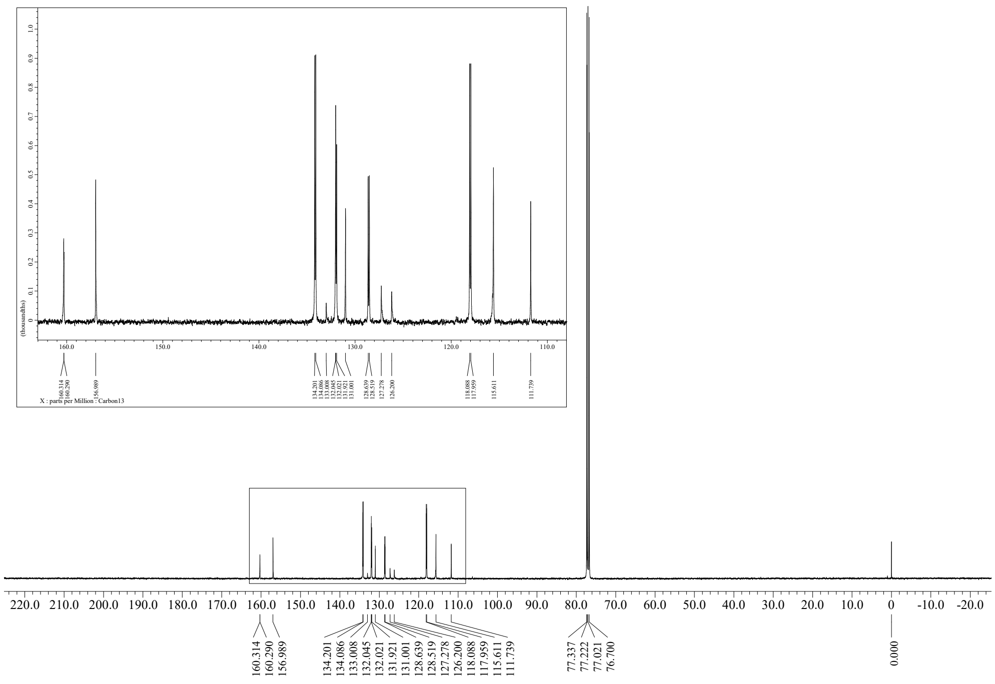

Figure S30. ^{13}C NMR spectrum of **P7** in $CDCl_3$.

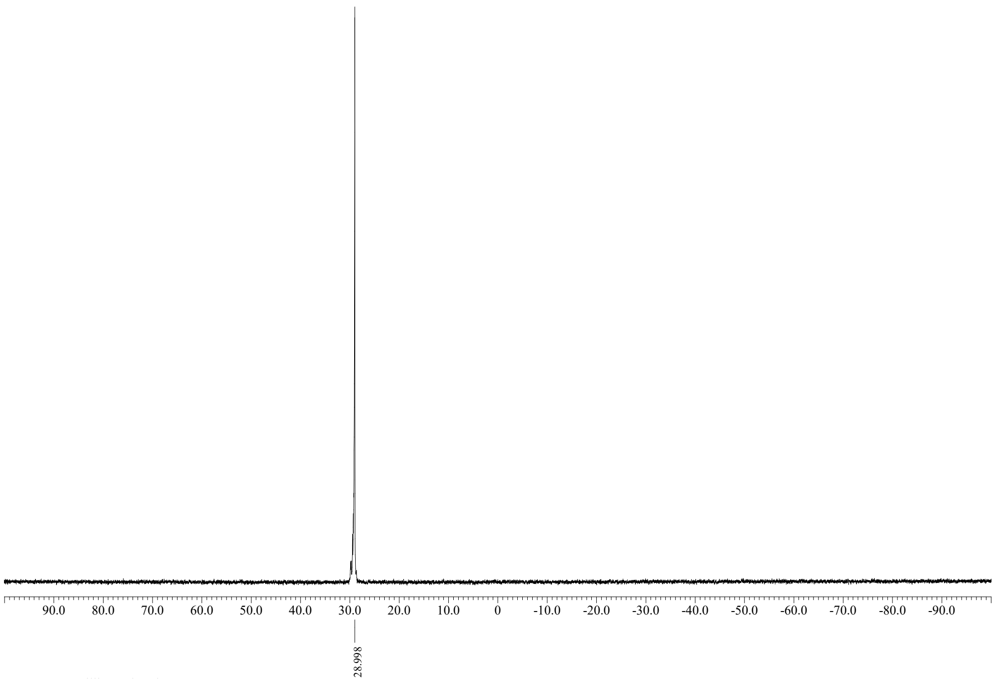

Figure S31. ^{31}P NMR spectrum of **P7** in $CDCl_3$.

P8

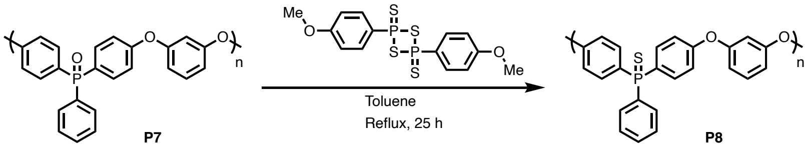

Scheme S9. Synthesis of **P8**.

According to the procedure B, **P7** (0.394 g, 1.03 mmol, 1 eq.) and Lawesson's reagent (0.384 g, 0.949 mmol, 0.921 eq.) were reacted in toluene (10 mL) under reflux for 25 h to give the polymer (**P8**). The yield was 0.345 g (83.6% yield).

M_n = 28700, M_w = 51300 (SEC in NMP with LiBr).

^{1}H NMR (400 MHz, $CDCl_3$) δ 7.74–7.63 (6H), 7.52–7.41 (3H), 7.33 (t, $^{3}J_{H–H}$ = 8.2 Hz, 1H), 7.04–7.01 (4H), 6.84 (dd, $^{3}J_{H–H}$ = 8.2 Hz, J = 1.9 Hz, 2H), 6.78 (t, $^{4}J_{H–H}$ = 1.9 Hz, 1H) ppm.

$^{13}C\{^{1}H\}$ NMR (101 MHz, $CDCl_3$) δ 160.0 (d, $^{4}J_{C–P}$ = 2.9 Hz), 156.9, 134.3 (d, $J_{C–P}$ = 12.0 Hz), 132.9 (d, $^{1}J_{C–P}$ = 86.2 Hz), 132.1 (d, $J_{C–P}$ = 10.6 Hz), 131.7 (d, $^{4}J_{C–P}$ = 2.4 Hz), 131.0, 128.6 (d, $J_{C–P}$ = 12.5 Hz), 127.0 (d, $^{1}J_{C–P}$ = 89.6 Hz), 117.9 (d, $J_{C–P}$ = 13.5 Hz), 115.7, 111.7 ppm.

$^{31}P\{^{1}H\}$ NMR (162 MHz, $CDCl_3$) δ 42.4 (s) ppm.

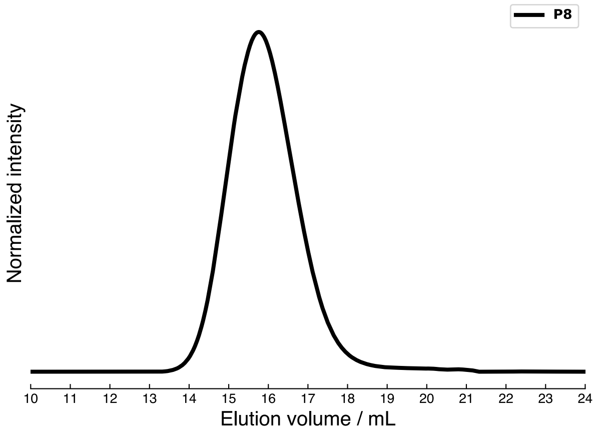

Figure S32. SEC traces of **P8** in NMP with LiBr (10 mM).

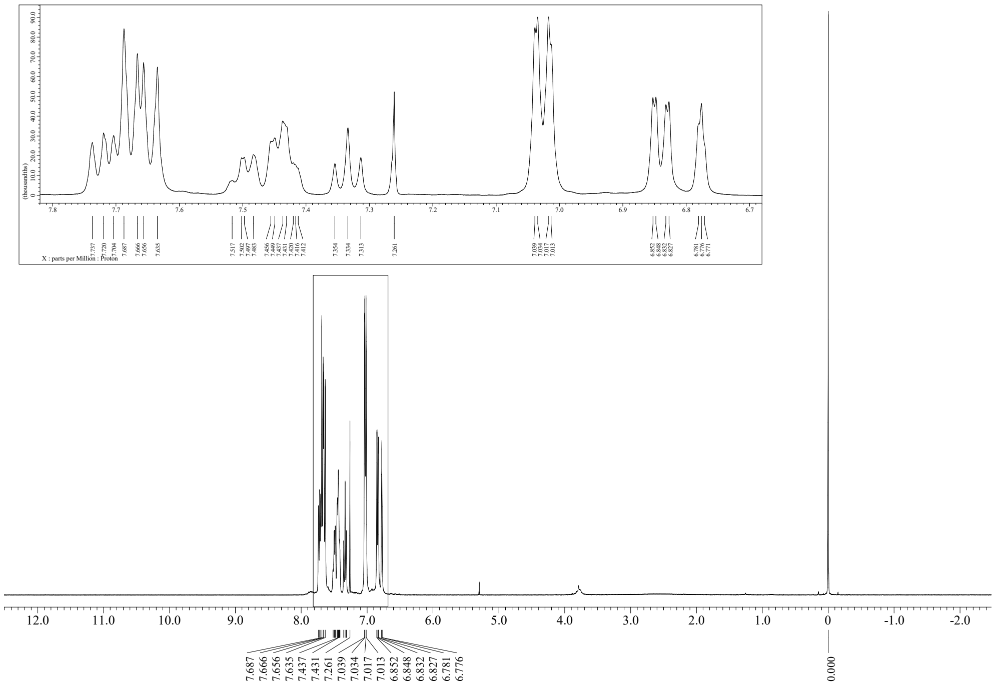

Figure S33. ^{1}H NMR spectrum of **P8** in $CDCl_3$.

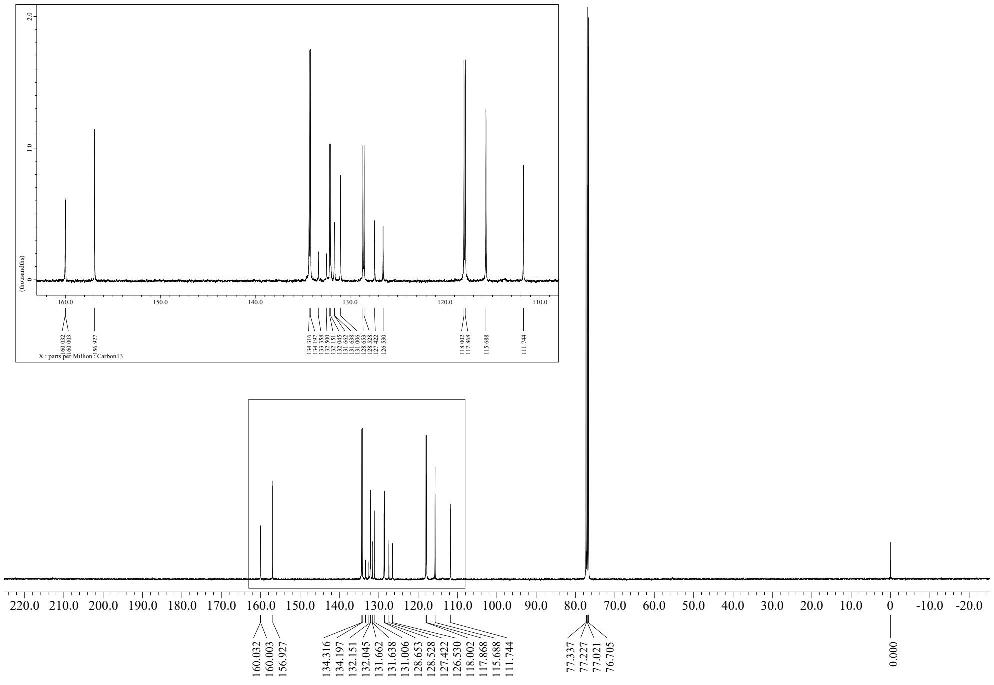

Figure S34. ^{13}C NMR spectrum of **P8** in $CDCl_3$.

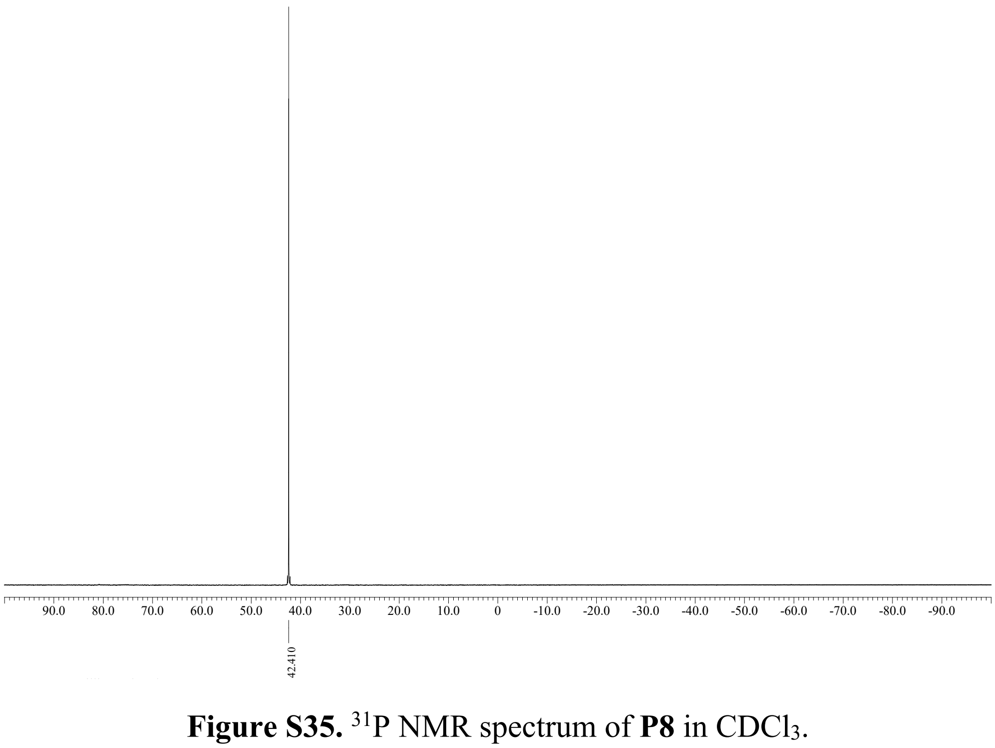

Figure S35. ^{31}P NMR spectrum of **P8** in $CDCl_3$.

P9

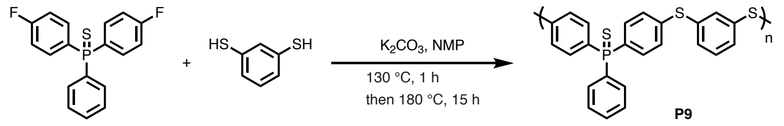

Scheme S10. Synthesis of **P9**.

According to the procedure A, bis(4-fluorophenyl)phenylphosphine sulfide (0.990 g, 3.00 mmol, 1 eq.) and 1,3-dimercaptobenzene (0.420 g, 2.95 mmol, 0.983 eq.) were reacted in the presence of K_2CO_3 (0.829 g, 6.00 mmol, 2.00 eq.) in NMP (5 mL) at 130 °C for 1 h and then at 180 °C for 15 h to give the polymer (**P9**). The yield was 0.819 g (63.4% yield).

M_n = 17400, M_w = 77200 (SEC in $CHCl_3$).

^{1}H NMR (400 MHz, $CDCl_3$) δ 7.67 (dd, J = 13.4 Hz, J = 7.2 Hz, 2H), 7.58–7.53 (5H), 7.50–7.47 (1H), 7.43–7.31 (5H), 7.22–7.21 (4H) ppm.

^{13}C{^{1}H} NMR (101 MHz, $CDCl_3$) δ 141.8 (d, $^4J_{C\text{–}P}$ = 2.9 Hz), 137.2, 134.3, 133.2, 132.8 (d, $J_{C\text{–}P}$ = 11.1 Hz), 132.1 (d, $J_{C\text{–}P}$ = 11.1 Hz), 131.9,* 131.8 (d, $^4J_{C\text{–}P}$ = 2.4 Hz), 130.7, 130.5 (d, $^1J_{C\text{–}P}$ = 87.2 Hz), 128.6 (d, $J_{C\text{–}P}$ = 12.5 Hz), 128.3 (d, $J_{C\text{–}P}$ = 13.0 Hz) ppm. *The peak at 131.9 ppm should have a coupling partner peak at approximately 132.7 ppm, which, however, overlaps with other peaks and cannot be observed.

^{31}P{^{1}H} NMR (162 MHz, $CDCl_3$) δ 42.9 (s) ppm.

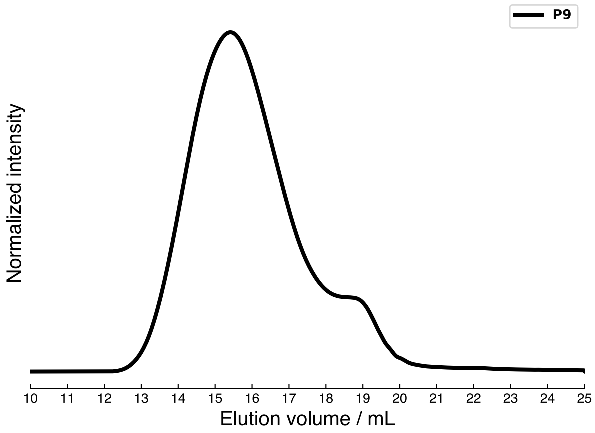

Figure S36. SEC traces of **P9** in $CHCl_3$.

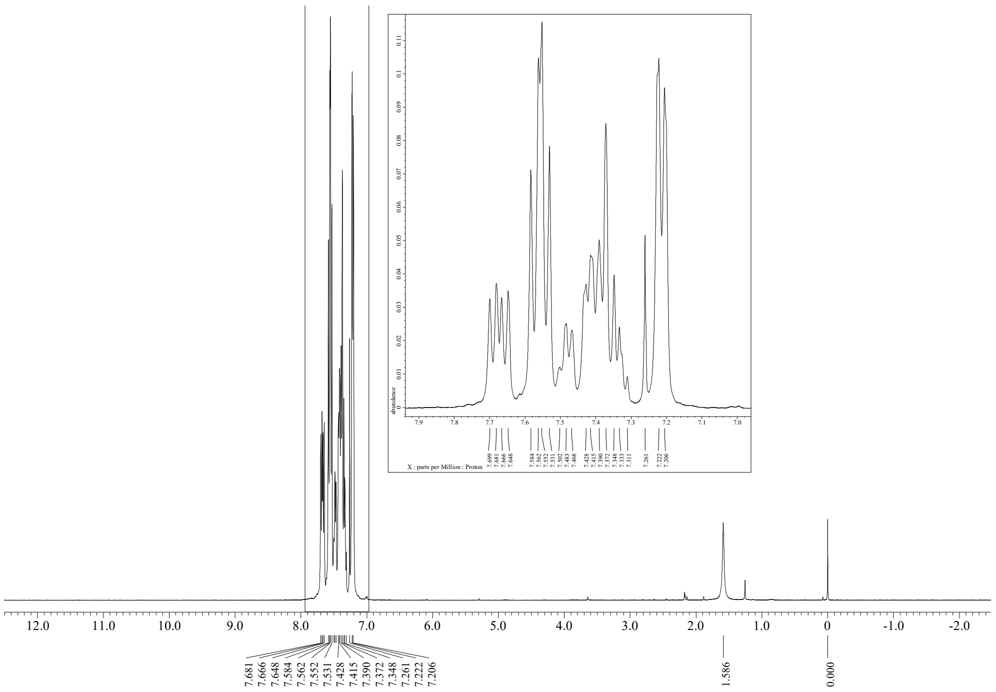

Figure S37. ^{1}H NMR spectrum of **P9** in $CDCl_3$.

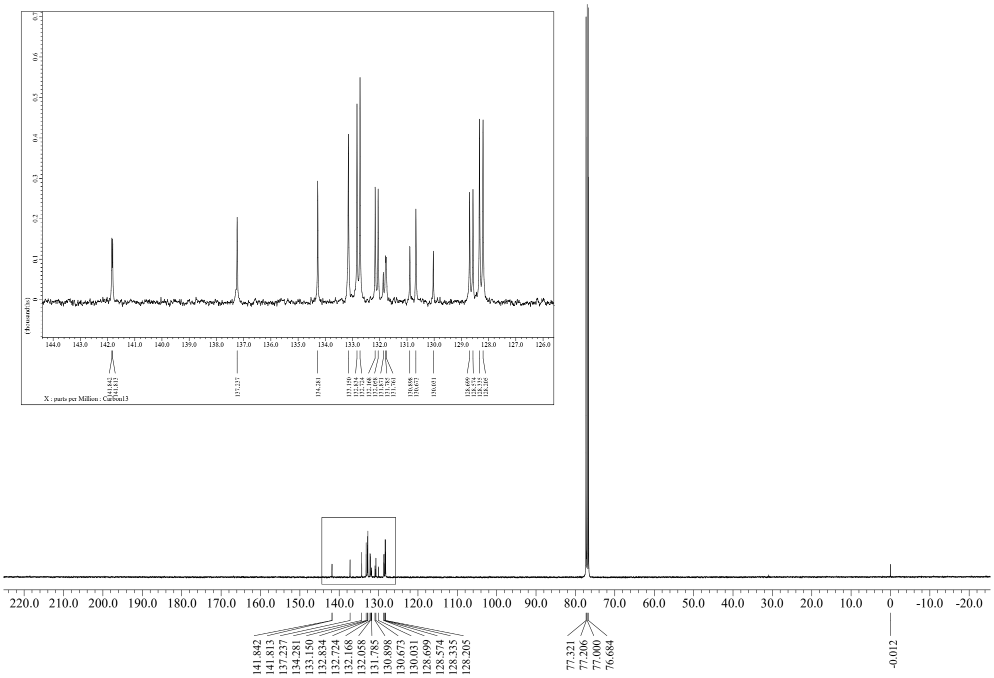

Figure S38. ^{13}C NMR spectrum of **P9** in $CDCl_3$.

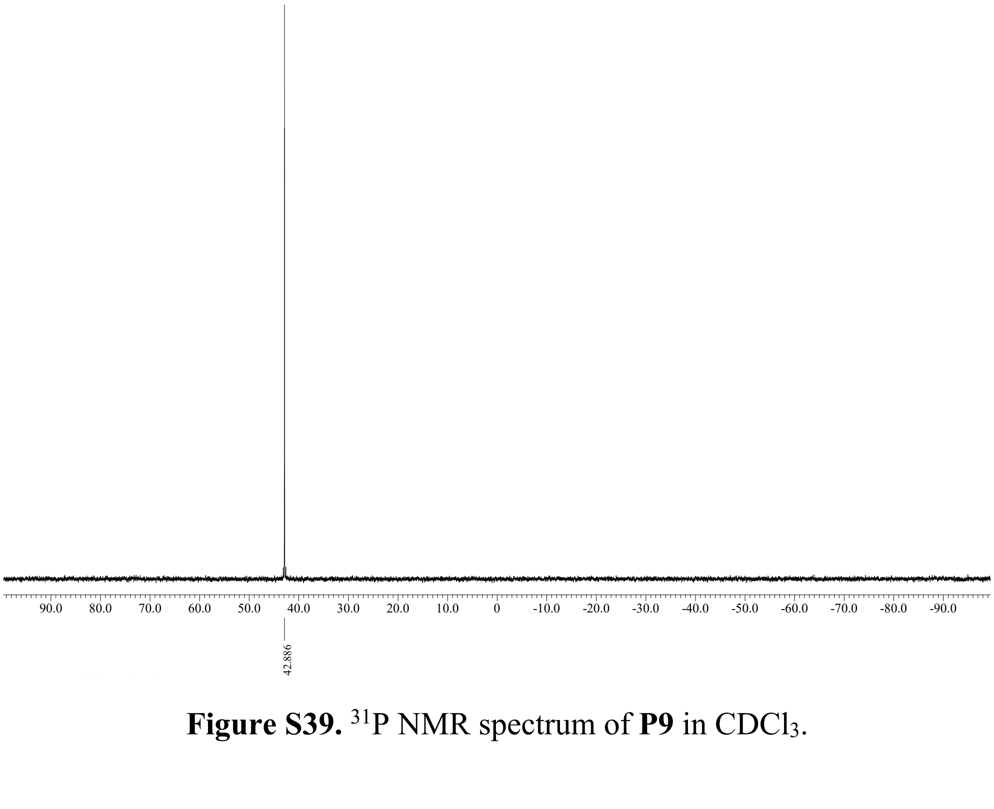

Figure S39. ^{31}P NMR spectrum of **P9** in $CDCl_3$.

P10

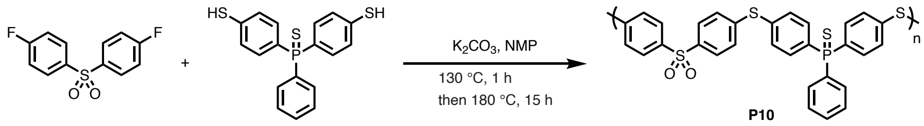

Scheme S11. Synthesis of **P10**.

According to the procedure A, bis(4-fluorophenyl) sulfone (0.236 g, 0.928 mmol, 1 eq.) and bis(4-mercaptophenyl)phenylphosphine sulfide (0.327 g, 0.912 mmol, 0.983 eq.) were reacted in the presence of K_2CO_3 (0.304 g, 2.20 mmol, 2.37 eq.) in NMP (2 mL) at 130 °C for 1 h and then at 180 °C for 15 h to give the polymer (**P10**). The yield was 0.478 g (90.8% yield).

M_n = 10600, M_w = 18000 (SEC in $CHCl_3$).

^{1}H NMR (400 MHz, $CDCl_3$) δ 7.81 (d, $^3J_{H-H}$ = 8.1 Hz, 4H), 7.73–7.60 (6H), 7.54–7.52 (1H), 7.48–7.46 (2H), 7.41–7.35 (8H) ppm.

^{13}C{^{1}H} NMR (101 MHz, $CDCl_3$) δ 142.7–142.6, 139.7–139.6, 138.2–138.0, 133.1 (d, J_{C-P} = 11.6 Hz), 133.0 (d, J_{C-P} = 11.6 Hz), 132.2–132.1, 131.52–131.47, 130.6 (d, J_{C-P} = 13.0 Hz), 130.3, 130.2, 128.9–128.7, 128.4 ppm.

^{31}P{^{1}H} NMR (162 MHz, $CDCl_3$) δ 42.9 (s) ppm.

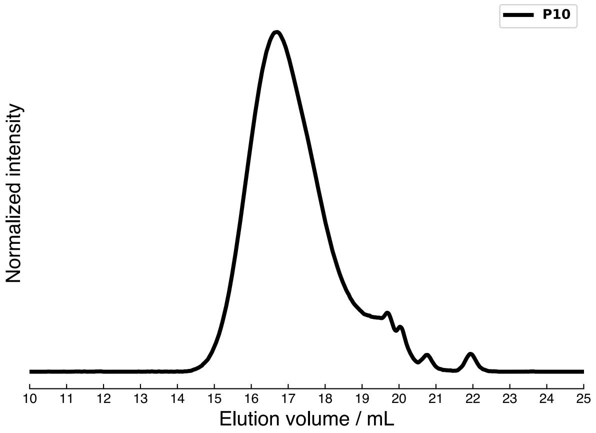

Figure S40. SEC traces of **P10** in $CHCl_3$.

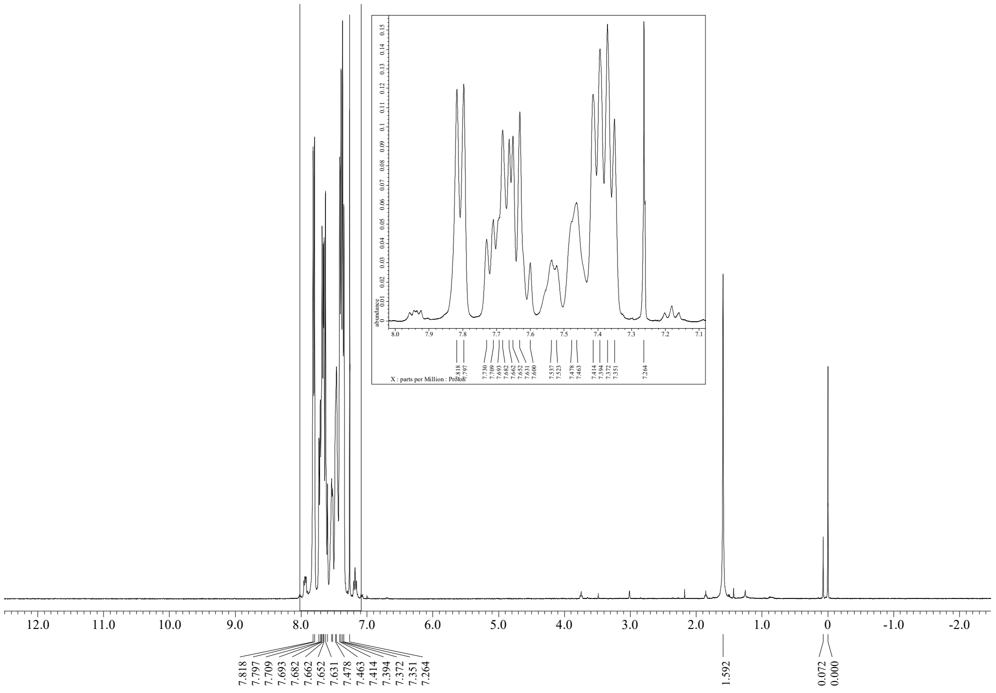

Figure S41. ^1H NMR spectrum of **P10** in $CDCl_3$.

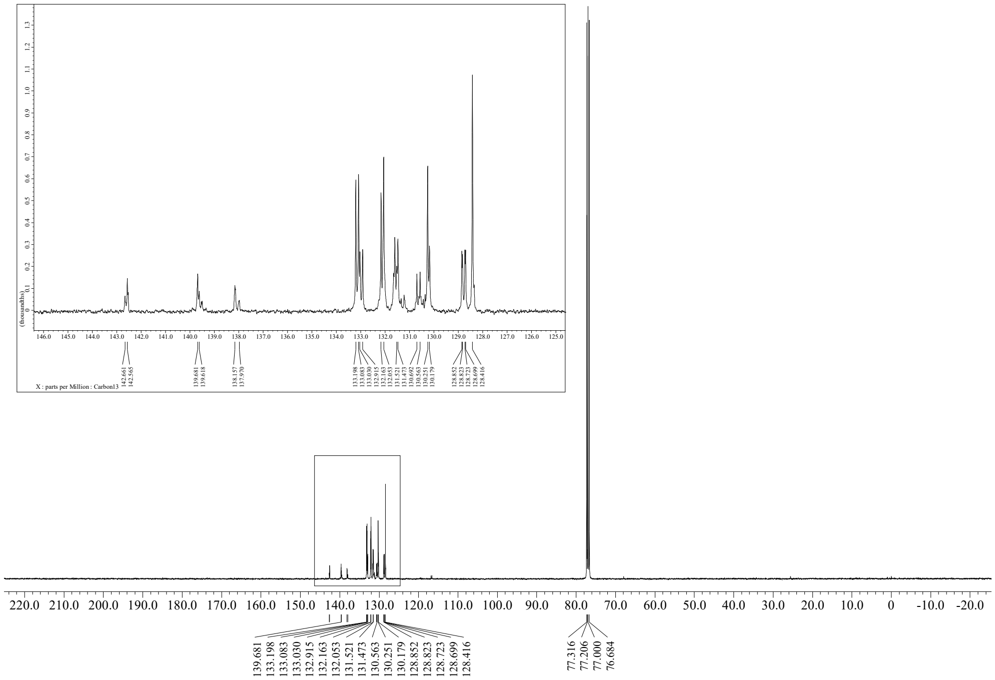

Figure S42. ^{13}C NMR spectrum of **P10** in $CDCl_3$.

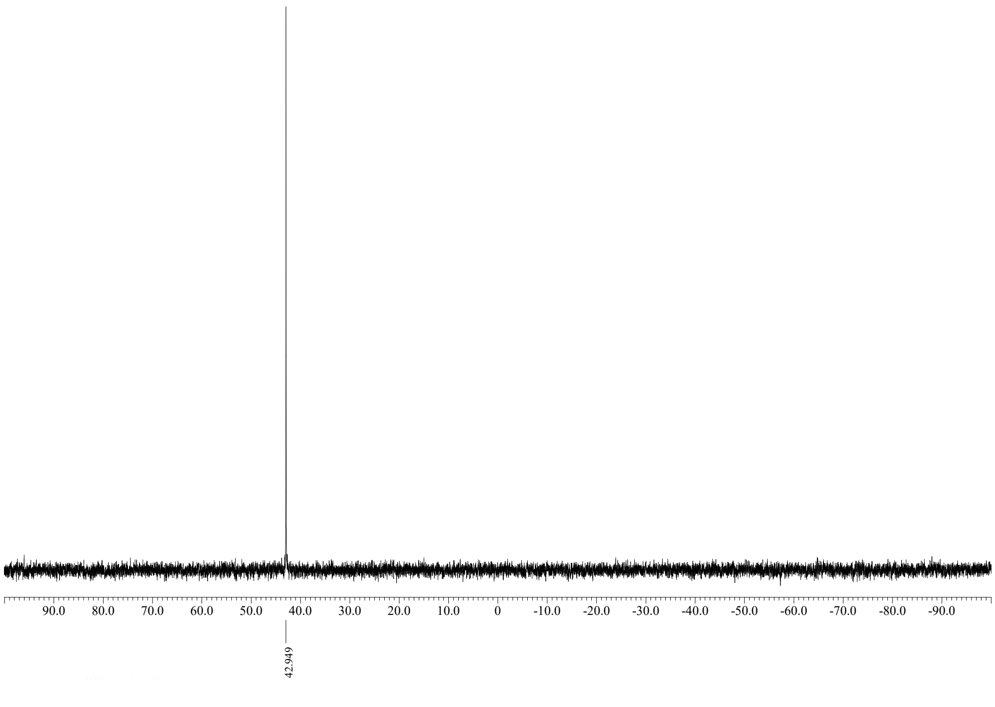

Figure S43. ^{31}P NMR spectrum of **P10** in $CDCl_3$.

P11

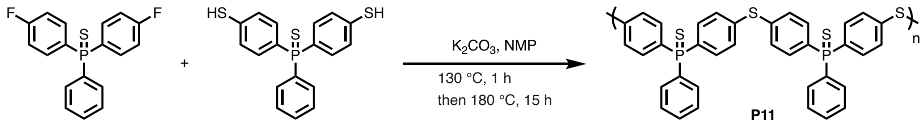

Scheme S12. Synthesis of **P11**.

According to the procedure A, bis(4-fluorophenyl)phenylphosphine sulfide (0.337 g, 1.02 mmol, 1 eq.) and bis(4-mercaptophenyl)phenylphosphine sulfide (0.358 g, 0.999 mmol, 0.979 eq.) were reacted in the presence of K_2CO_3 (0.304 g, 2.20 mmol, 2.16 eq.) in NMP (2 mL) at 130 °C for 1 h and then at 180 °C for 15 h to give the polymer (**P11**). The yield was 0.365 g (55.7% yield).

M_n = 7900, M_w = 17200 (SEC in $CHCl_3$).

^{1}H NMR (400 MHz, $CDCl_3$) δ 7.72–7.59 (6H), 7.54–7.50 (1H), 7.46–7.43 (2H), 7.38–7.36 (4H) ppm.

^{13}C{^{1}H} NMR (101 MHz, $CDCl_3$) δ 139.4 (d, $^{4}J_{C-P}$ = 2.9 Hz), 133.0 (d, J_{C-P} = 11.6 Hz), 132.1 (d, J_{C-P} = 10.6 Hz), 132.0 (d, $^{4}J_{C-P}$ = 2.4 Hz), 131.9 (d, $^{1}J_{C-P}$ = 86.7 Hz), 131.7 (d, $^{1}J_{C-P}$ = 86.2 Hz), 130.6 (d, J_{C-P} = 13.0 Hz), 128.7 (d, J_{C-P} = 13.0 Hz) ppm.

^{31}P{^{1}H} NMR (162 MHz, $CDCl_3$) δ 43.0, 42.7 ppm.

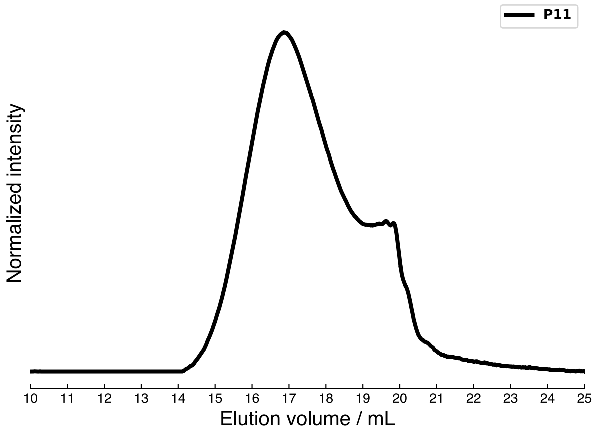

Figure S44. SEC traces of **P11** in $CHCl_3$.

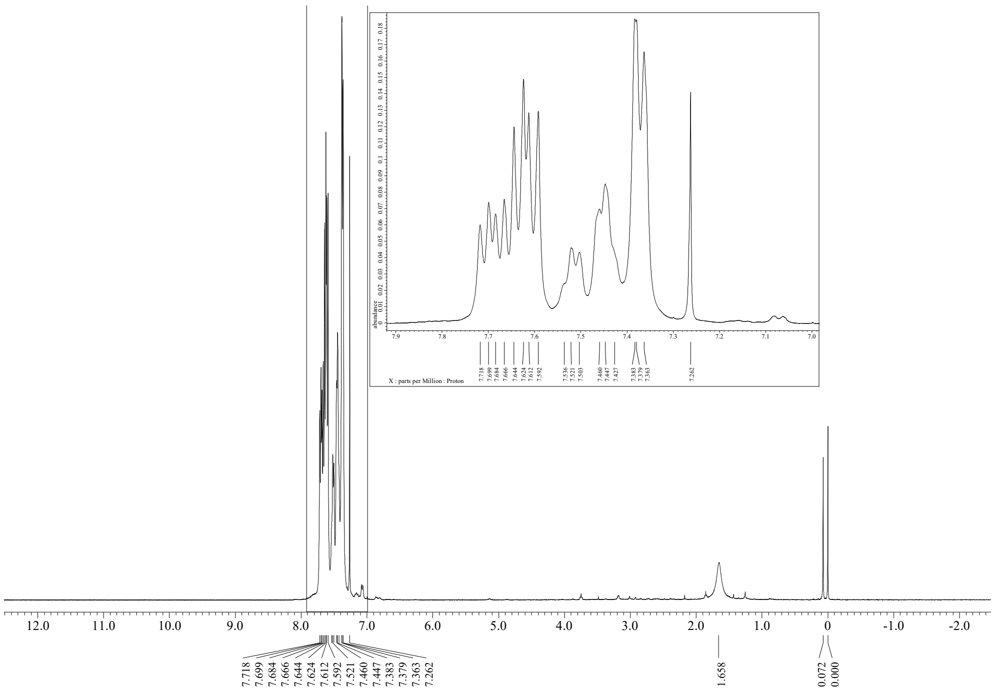

Figure S45. ^{1}H NMR spectrum of **P11** in $CDCl_3$.

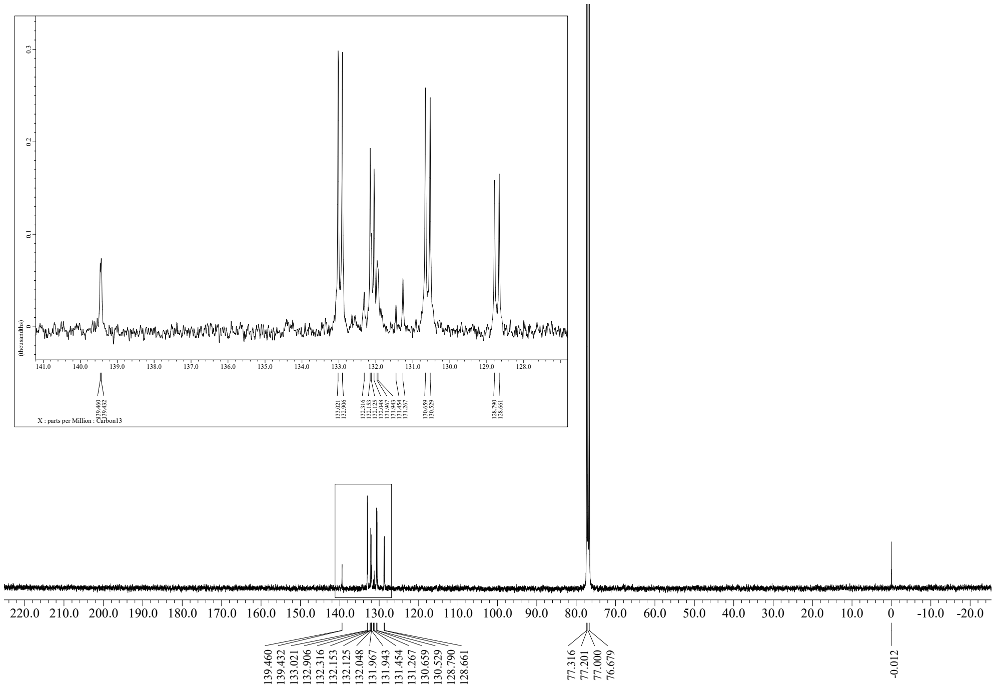

Figure S46. ^{13}C NMR spectrum of **P11** in $CDCl_3$.

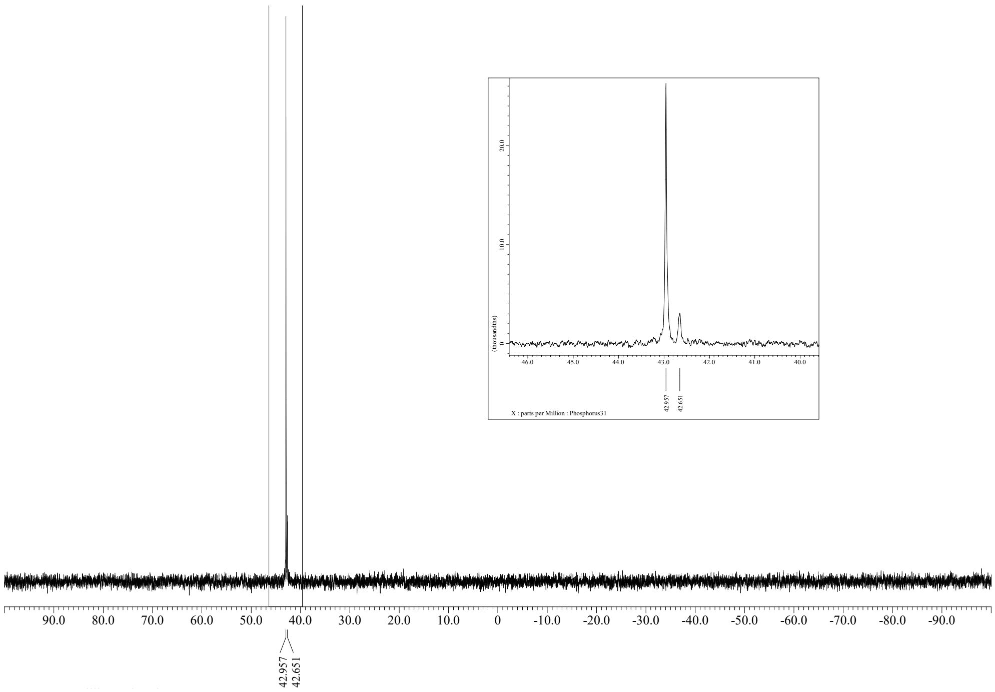

Figure S47. ^{31}P NMR spectrum of **P11** in $CDCl_3$.

The densities of the polymers (**P1**–**P6**) were determined using a pycnometer with their films (Table S1). Values represent the average of three independent measurements (mean ± standard deviation).

Table S1. Densities of polymers (**P1**–**P6**).

Polymer	**P1**	**P2**	**P3**	**P4**	**P5**	**P6**
Density/g cm^{-3}	1.27±0.04	1.21±0.01	1.27±0.04	1.21±0.06	1.30±0.07	1.21±0.03

4. Stability Tests

The resistance of the P=S group toward moisture and light irradiation was examined. A solution of **P6** in THF/H_2O (9/1, v/v) was stirred under air at 60 °C for 24 h, and no change was observed in the ^{31}P NMR spectrum (Figure S48b). Irradiation of a THF solution of **P6** with UV light at 254 nm for 1 h also resulted in no detectable change in the ^{31}P NMR spectrum (Figure S48c).

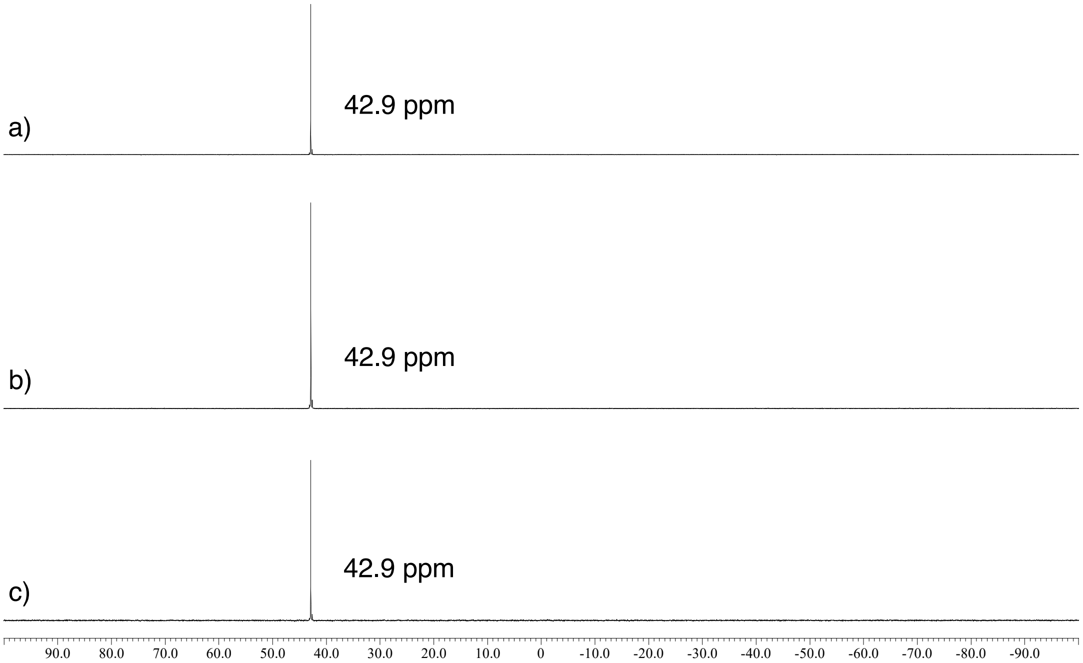

Figure S48. Stacked ^{31}P NMR spectra of a) pristine **P6**, b) the sample obtained after heating **P6** at 60 °C for 24 h in THF/H_2O (9/1, v/v), and c) the sample obtained after irradiating **P6** with UV light at 254 nm for 1 h in THF.

5. Optical Properties

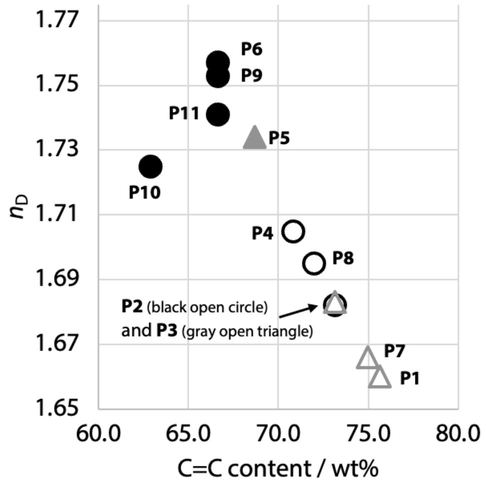

Figure S49. Relationship between the C=C content and the n_D values of **P1–P11**. Gray open triangles represent the poly(ether)s containing the P=O groups (**P1**, **P3**, and **P7**), a gray solid triangle represents the poly(thioether) containing the P=O groups (**P5**), black open circles represent the poly(ether)s containing the P=S groups (**P2**, **P4**, and **P8**), and black solid circles represent the poly(thioether)s containing the P=S groups (**P6** and **P9–P11**).

The Abbe number (ν_D) can be expressed using the refractive index at 589 nm (n_D), molar refractivity at 589 nm ($[R_D]$), and molar dispersion ($[\Delta R]$):[5]

$$\nu_D = \frac{6n_D}{(n_D{}^2+2)(n_D+1)} \frac{[R_D]}{[\Delta R]} \quad (1)$$

The $[\Delta R]$ value is defined as the difference between molar refractivities at 486 and 656 nm ($[R_F]$ and $[R_C]$, respectively), and the $[R_D]$, $[R_F]$, and $[R_C]$ values can be calculated from the polarizability (α) at the corresponding wavelengths and the Avogadro constant (N_A):

$$[\Delta R] = [R_F] - [R_C] \quad (2)$$

$$[R_X] = \frac{4}{3}\pi N_A \alpha_X \quad (X = D, F, \text{or } C) \quad (3)$$

The α values for three model compounds (**Model_S**, **Model_SO$_2$**, and **Model_P=S**, Figure S50) representing partial structures of **P6**, **P10** and **P11** were calculated using density functional theory, and the corresponding [R_D], [R_F], [R_C], and [R_D]/[ΔR] values are summarized in Table S2. The calculated [R_D]/[ΔR] value of **Model_P=S** (17.2) was found to be larger than those of **Model_S** (15.5) and **Model_SO$_2$** (15.7). Since the Abbe number is proportional to the [R_D]/[ΔR] value, this result supports the experimental observation that **P11** exhibits a higher Abbe number than expected from the general trend.

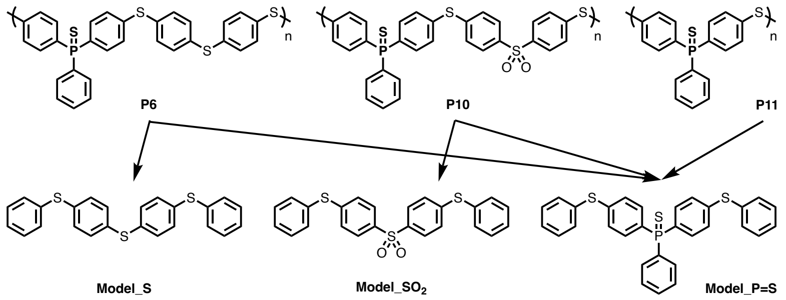

Figure S50. Structures of **Model_S**, **Model_SO$_2$**, and **Model_P=S**.

Table S2. Calculated molar refractivities ([R_F], [R_D], and [R_C]) and [R_D]/[ΔR] values.

Model compound	[R_F] (cm^3 mol^{-1})	[R_D] (cm^3 mol^{-1})	[R_C] (cm^3 mol^{-1})	[R_D]/[ΔR]
**Model_S**	157.97	150.80	148.27	15.5
**Model_SO$_2$**	157.80	150.69	148.19	15.7
**Model_P=S**	196.90	188.85	185.94	17.2

6. References

1 Frisch, M. J.; Trucks, G. W.; Schlegel, H. B.; Scuseria, G. E.; Robb, M. A.; Cheeseman, J. R.; Scalmani, G.; Barone, V.; Petersson, G. A.; Nakatsuji, H.; Li, X.; Caricato, M.; Marenich, A. V.; Bloino, J.; Janesko, B. G.; Gomperts, R.; Mennucci, B.; Hratchian, H. P.; Ortiz, J. V.; Izmaylov, A. F.; Sonnenberg, J. L.; Williams-Young, D.; Ding, F.; Lipparini, F.; Egidi, F.; Goings, J.; Peng, B.; Petrone, A.; Henderson, T.; Ranasinghe, D.; Zakrzewski, V. G.; Gao, J.; Rega, N.; Zheng, G.; Liang, W.; Hada, M.; Ehara, M.; Toyota, K.; Fukuda, R.; Hasegawa, J.; Ishida, M.; Nakajima, T.; Honda, Y.; Kitao, O.; Nakai, H.; Vreven, T.; Throssell, K.; Montgomery, J. A., Jr.; Peralta, J. E.; Ogliaro, F.; Bearpark, M. J.; Heyd, J. J.; Brothers, E. N.; Kudin, K. N.; Staroverov, V. N.; Keith, T. A.; Kobayashi, R.; Normand, J.; Raghavachari, K.; Rendell, A. P.; Burant, J. C.; Iyengar, S. S.; Tomasi, J.; Cossi, M.; Millam, J. M.; Klene, M.; Adamo, C.; Cammi, R.; Ochterski, J. W.; Martin, R. L.; Morokuma, K.; Farkas, O.; Foresman, J. B.; Fox, D. J. *Gaussian 16 Revision C.01*, Gaussian, Inc., Wallingford CT, 2019.

2 Hifumi, R.; Tomita, I. High Refractive and Low Birefringent Materials Based on Poly(Arylene Ether Phosphine Oxide)s and Poly(Arylene Ether Phosphine Sulfide)s. *Polymer* **2020**, *186*, 121855. https://doi.org/10.1016/j.polymer.2019.121855.

3 Hifumi, R.; Ochiai, K.; Tomita, I. Synthesis of Phosphine Sulfide Group-Containing Aromatic Poly(Ether)s with Aliphatic Substituents on the Phosphorus Atoms and Low Dielectric Properties. *Polym. J.* **2024**, *56*, 997–1004. https://doi.org/10.1038/s41428-024-00940-7.

4 Hifumi, R.; Tomita, I. Synthesis and Dielectric Properties of Aromatic Poly(thioether)s with Triphenylphosphine Sulfide Moieties. *J. Netw. Polym. Jpn.* **2024**, *45*, 143–150. https://doi.org/https://doi.org/10.11364/networkedpolymer.45.3_143. (in Japanese).

5 Watanabe, S.; Oyaizu, K. Designing Strategy for High Refractive Index Polymers: From the Molecular Level to Bulk Structure Control. *Bull. Chem. Soc. Jpn.* **2023**, *96*, 1108–1128. https://doi.org/10.1246/bcsj.20230177.
